# Band structure database of layered intercalation compounds with various intercalant atoms and layered hosts

**DOI:** 10.1038/s41597-024-04008-2

**Published:** 2024-11-18

**Authors:** Naoto Kawaguchi, Kiyou Shibata, Teruyasu Mizoguchi

**Affiliations:** 1 Department of Materials Science and Engineering, The University of Tokyo, 7-3-1 Hongo, Bunkyo, Tokyo 113-8656 Japan; 2 Institute of Industrial Science, The University of Tokyo, 4-6-1 Komaba, Meguro, Tokyo 153-8505 Japan

**Keywords:** Electronic structure, Electronic properties and materials

## Abstract

Here we provide a database comprising electronic band structures of 9,004 layered intercalation compounds, where atoms are intercalated into a host layered compound with different intercalant atoms, along with 468 structures related to the layered host compounds. Additionally, we provide properties derived from the electronic states such as band gap as well as stability-related properties like formation energies. Direct comparison of the band structures before and after intercalation is generally challenging due to changes in their space group and *k*-path. However, in this study, we developed new *k*-paths consistent with the host materials, allowing for the direct comparison of band structures before and after intercalation. This enables direct and quantitative discussion of the band structure changes induced by the intercalations and provides a valuable database for intercalant-driven band engineering. Layered intercalation compounds are widely used in many fields, including superconductivity and energy applications, and understanding of electronic structures is necessary. The feature of our database holds promises for the development of layered compounds with enhanced functionalities through database utilization.

## Background & Summary

Layered intercalation compounds, characterized by structures where atoms or intercalants are inserted between layers of materials, have garnered considerable research interest due to their unique electronic states such as superconductivity^1–4^, spin glass^5^, and topological electronic states^6^ arising from their two-dimensional nature. Furthermore, they are also utilised as energy materials. For instance, LiC_6_ generated by Li intercalation into graphite is widely employed as the anode material in lithium-ion batteries^7,8^, while LiCoO_2_ serves as the cathode material^9^, owing to their ability to be intercalated and de-intercalated ions. Various synthesis methods including zero-valent metal intercalation^10^, liquid alloy method^11^, molten salt method^12^, molecular beam epitaxy (MBE) and chemical vapor deposition (CVD) method^13^, have been developed in recent years, leaving room for further exploration of new materials. However, it is not clear what combinations of intercalants and layered materials are stable and how the band structure of layered materials is changed by intercalants, and no methodology has been established for the design of interlayer compounds with the desired electronic structure. For instance, in the case of Graphite Intercalation Compounds (GIC), which are representative interlayer compounds, the phenomenon that only Na intercalation does not occur among alkali metals has been extensively studied by investigating electronic states in the field of computational science^14–19^. We have also reported the potential occurrence of charge density wave structures in GIC through electronic state analysis^20^ and demonstrated the applicability of linear regression equation derived from the hard and soft acids and bases principle in expressing the stability of about 7,000 layered intercalation compounds^21^. Despite these intensive studies, the general physics of how intercalation and intercalant species affect the band structure of hosts has not been established.

Furthermore, in the present research on the electronic states utilizing databases, comprehensive databases of inorganic compounds such as Materials Project (MP)^22^ and Open Quantum Materials Database^23,24^ exist, containing band structure data. Intensive research is being conducted using these databases. For example, there have been reports on the design of useful band structures for thermoelectric materials by screening based on elemental and local structures^25^ and on the prediction of band gaps for binary compounds in Materials Project at the HSE06 level from PBE calculation by a machine learning method^26^. Moreover, databases specialized in specific structures, such as 2D materials^27–29^, perovskites^30^, and bilayer materials^31^ have also been constructed. There have been reports of the use of such specific databases, for example, in 2D-materials, the band structures with GW-level accuracy were successfully predicted^32^. Thus, while various databases on crystalline materials are being developed, no database has yet been constructed on the layered intercalation compounds formed by the intercalation of atoms into the layered materials despite their importance. Therefore, the effect of intercalants on the electronic band structure is not clear.

Based on the above background, in this study, a database of formation energies, reaction energies resulting from intercalation (intercalation energies), and electronic band structures for layered intercalation compounds has been constructed. Specifically, we systematically calculated the optimized structures and electronic states of 9,024 layered intercalation compounds and band structures were computed for 9,004 structures where the space groups remained unchanged with structural optimization. Additionally, electronic band structures of corresponding host layered structures (188 intercalant-erased structures, 142 bulk structures and 142 slab model structures) were also computed, resulting in a total of 9,476 electronic band structures.

Direct comparison of band structures between layered intercalation compounds and their host materials poses challenges due to potential changes in their structural symmetry caused by the intercalation. To address this issue, we developed a new method for describing electronic band structures of layered intercalation compounds consistent with the host *k*-path. This enabled the construction of a database facilitating direct comparison of their band structure alterations induced by the intercalation. This method is expected to enable data-driven material design in fields where physical properties can be controlled by electronic states, such as energy storage materials, electronics materials and superconducting materials. For example, in storage materials, charge-discharge occurs by intercalation, and conductivity and electron mobility due to intercalation are discussed according to the band structure before and after intercalation^33^. The band structure of before/after intercalation can be easily compared using this database. Such knowledge of changes in band structure by intercalation is also expected to accelerate a series of studies attempting to control the band structure of layered materials by intercalation^34–36^. Furthermore, GICs exhibit superconductivity in multiple intercalants, and several previous studies have pointed out that superconductivity appears when free electrons enter a band with dispersion perpendicular to the GIC layers, called the interlayer band, as an important electronic band for the appearance of superconductivity^37–39^. However, GICs have crystal structures with different space groups, making direct comparisons difficult using conventional methods, and a method of comparing band structures by unifying structures that are not experimentally reported has been used^37,38^. This database facilitates the comparison of band structures for layered intercalation compounds with the same host but different crystal structures depending on the intercalant.

The number of layered intercalation compounds in this database, totalling 9,004 structures, is comparable to the number of materials in the 2D-materials databases (15,733^28^, 3,172^29^ and 6,351^27^ materials) and the perovskite database (5,329 materials^30^) mentioned above. Moreover, our database allows direct comparison of electronic band structures before and after intercalation, making it possible to quantitatively compare the band structure changes induced by the intercalation. This database also provides information that can be extracted from the band structure, such as band gaps, direct/indirect transitions and the presence of spin polarisation. Additionally, the database contains properties related to their stability, such as formation energy and intercalation energy. The utilization of this database is expected to enhance understanding of the electronic state changes induced by intercalants in the host materials, as well as facilitate research into materials exploration for new layered intercalation compounds and related substances.

## Methods

### Screening and structure generation of layered intercalation compounds

**Fig. 1:**
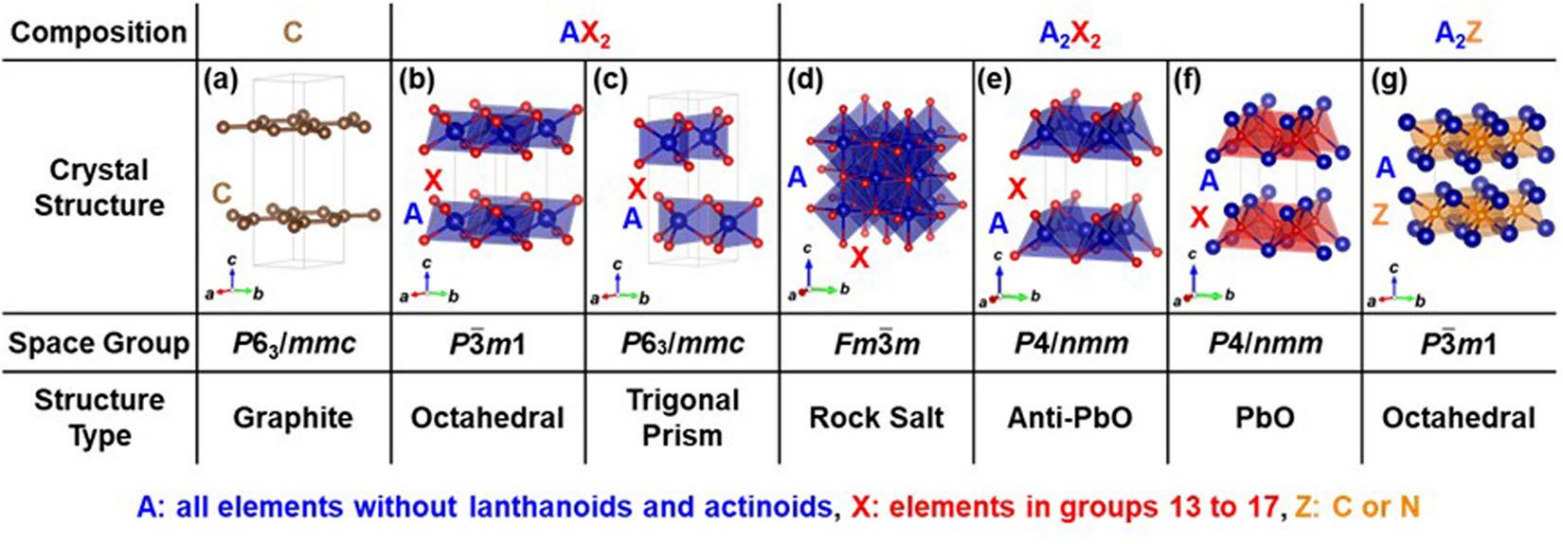
The crystal structures treated as host materials in this study. “A” represents all elements excluding lanthanoids and actinoids, “X” represents elements in groups 13 to 17 and “Z” represents carbon or nitrogen.

**Fig. 2:**
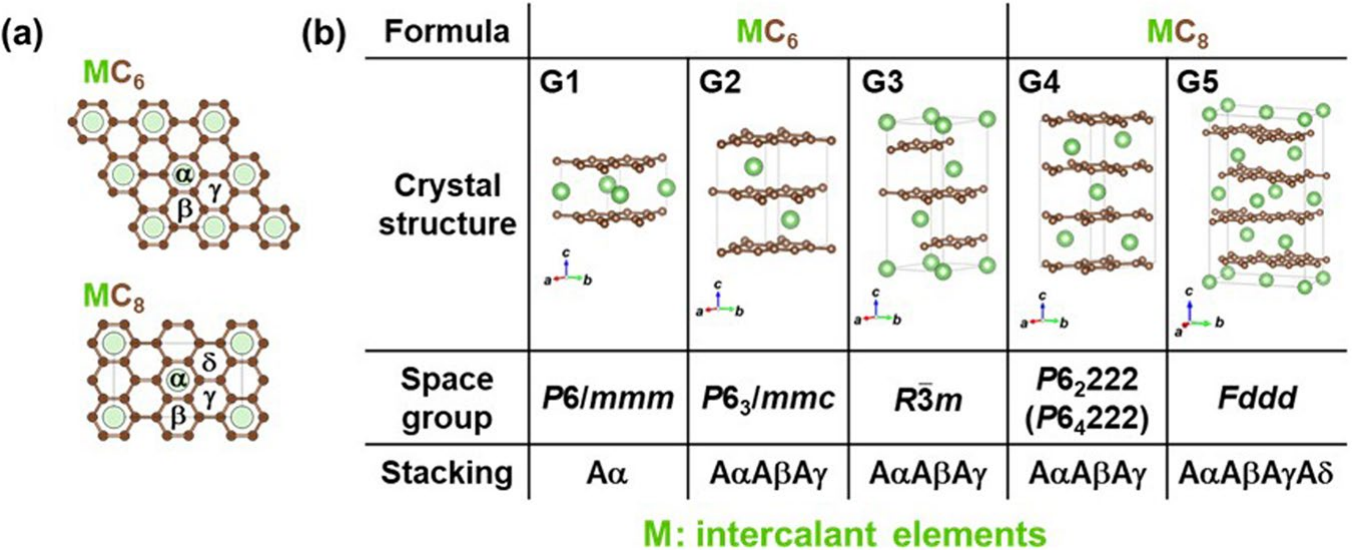
Structures of GICs. (**a**) Intercalation sites in compositions MC_6_, categorized into α, β and γ, and intercalation sites in compositions MC_8_, categorized into α, β, γ and *δ*. (**b**) Crystal structures of experimentally reported GICs. “M” represents intercalant elements. Symbols in the upper left of each crystal structure indicate the structure type assigned in this study.

**Fig. 3:**
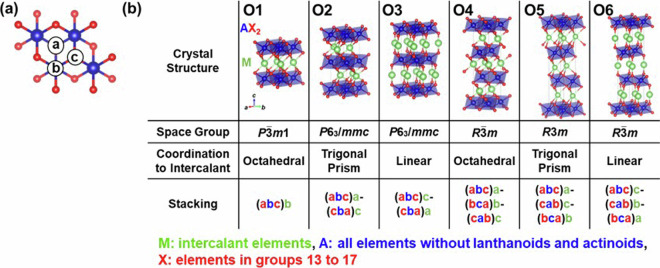
Structures of layered intercalation compounds represented by the composition MAX_2_ with the octahedral-type host. (**a**) Stacking sites of the elements. (**b**) Crystal structures with different coordination to intercalant and stacking order. Regarding the representation of the stacking, brackets indicate the host site, and the colors of the letters – blue, red, and green – represent the elements “A”, “X”, and “M”, respectively. Symbols in the upper left of each crystal structure indicate the structure type assigned in this study.

**Fig. 4:**
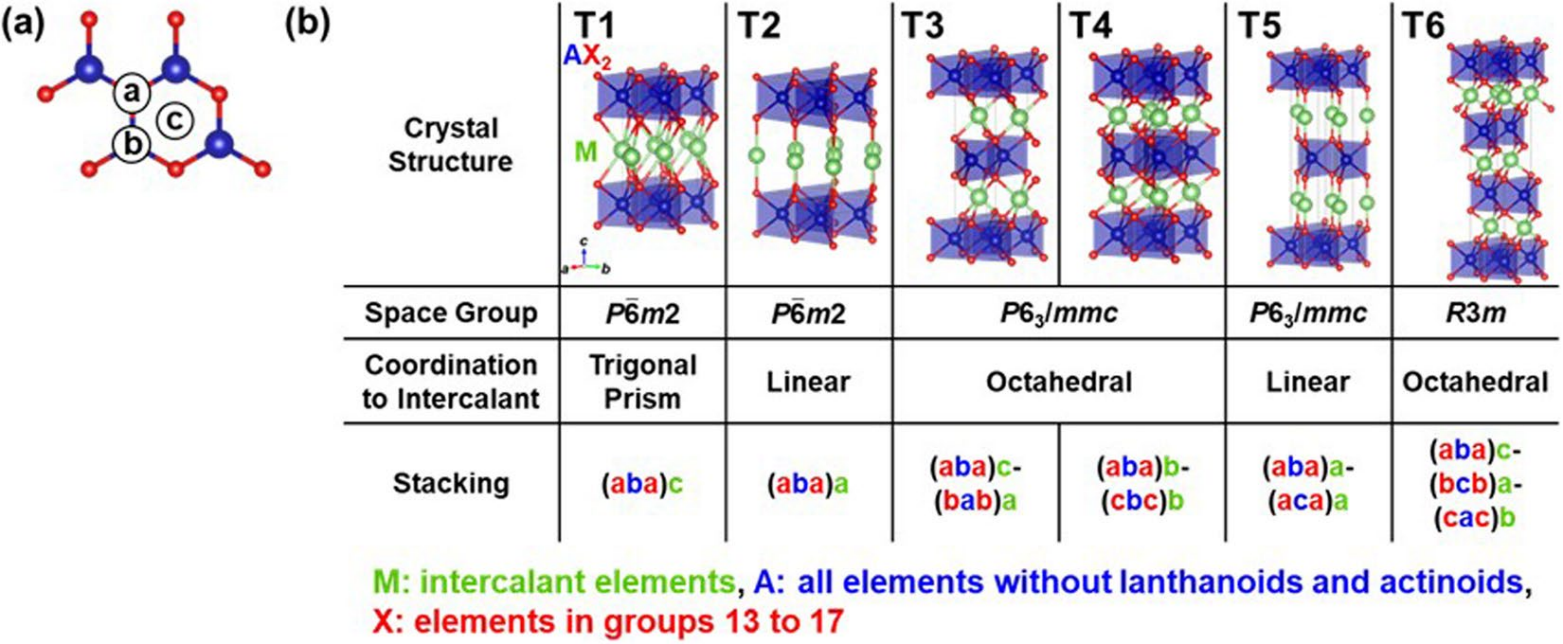
Structures of layered intercalation compounds represented by the composition MAX_2_ with the trigonal-prism-type host. (**a**) Stacking sites of the elements. (**b**) Crystal structures with different coordination to intercalant and stacking order. Regarding the representation of the stacking, brackets indicate the host site, and the colors of the letters – blue, red, and green – represent the elements “A”, “X”, and “M”, respectively. Symbols in the upper left of each crystal structure indicate the structure type assigned in this study.

**Fig. 5:**
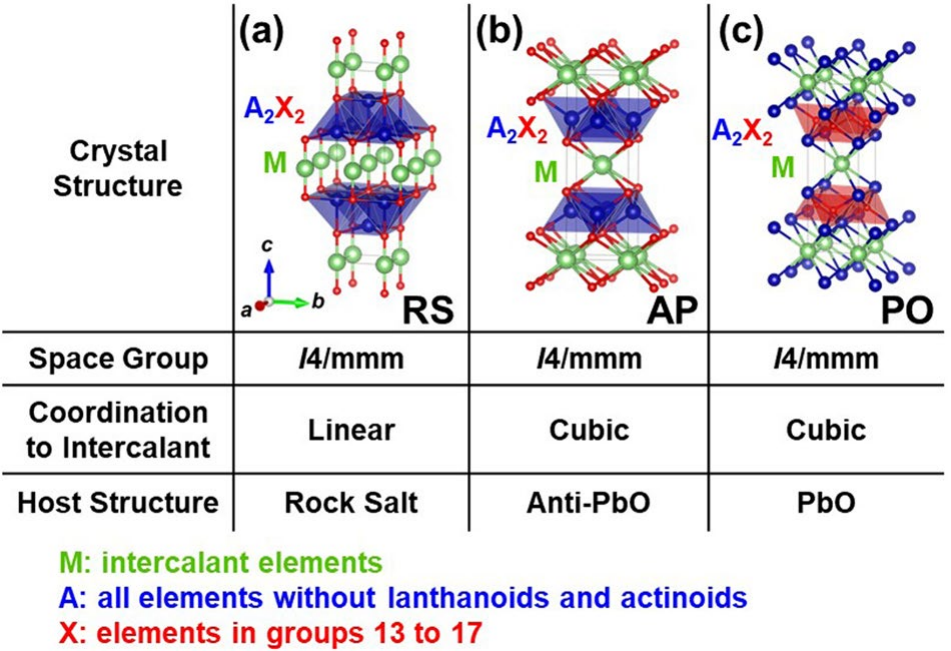
Structures of layered intercalation compounds represented by the composition MA_2_X_2_. (**a**) Crystal structure with a rock-salt-type host. (**b**) Crystal structure with an anti-PbO type host. (**c**) Crystal structure with a PbO type host. Symbols in the upper left of each crystal structure indicate the structure type assigned in this study.

**Fig. 6:**
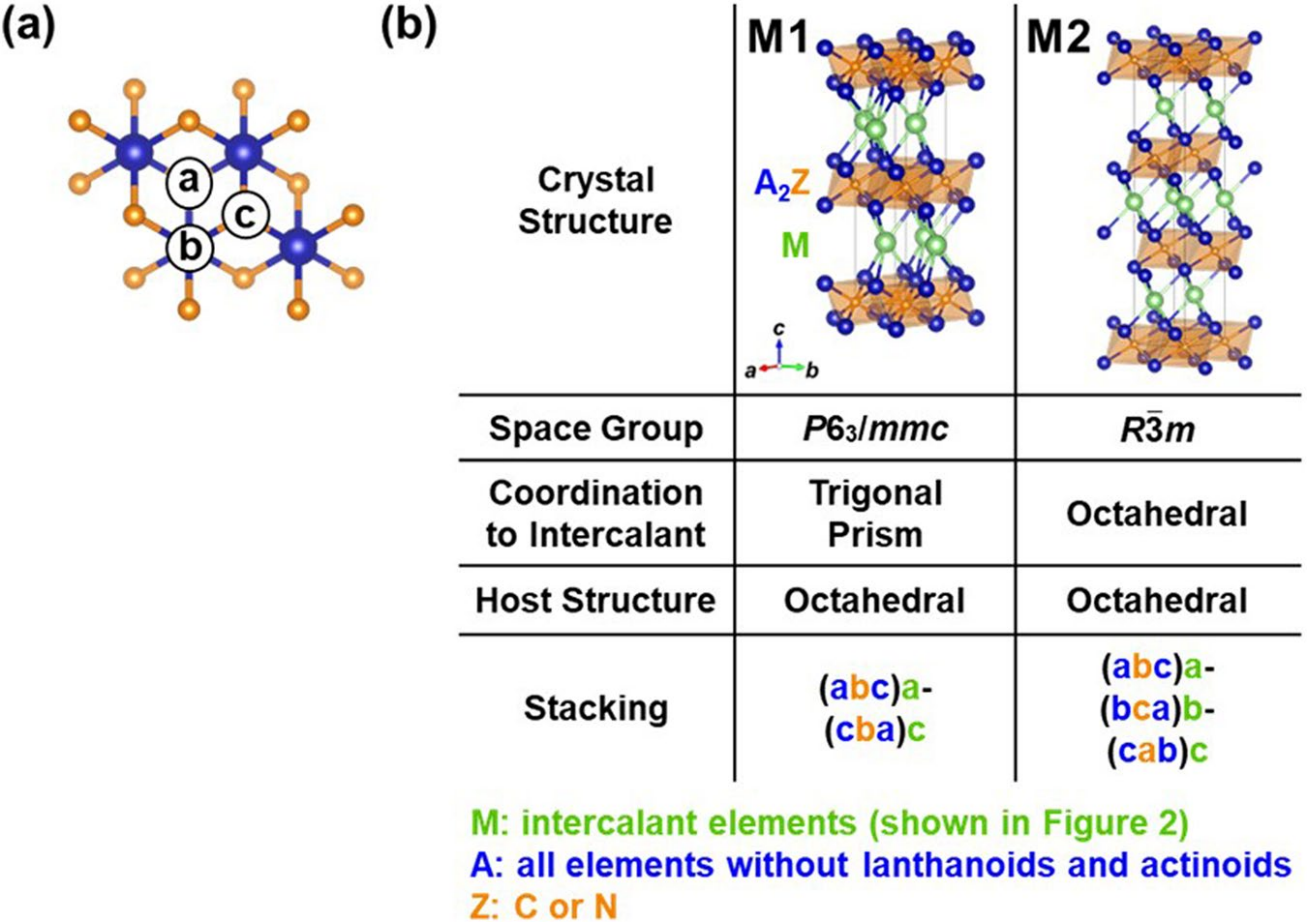
Structures of layered intercalation compounds represented by the composition MZA_2,_ known as MAX phase. (**a**) Stacking sites of the elements. (**b**) Experimentally reported crystal structures. Symbols in the upper left of each crystal structure indicate the structure type.

Basically, the structures of layered intercalation compounds were created using the similar method as in our previous study^21^. That is, seven types of structures were initially selected as hosts. The host crystal structures are shown in Fig. 1. Among the known structures, we have selected representative and well-known layered structures, including both the structure of the host alone and the structure with atoms intercalated into the host layers. Additionally, structures containing multiple layers, such as Cu_1-*x*_Bi_2_Se_3_^2^, were excluded, focusing solely on single-layer configurations. In the compositional formula of Fig. 1, “A” represents all elements excluding lanthanides and actinides, “X” represents elements from groups 13 to 17, and “Z” represents either C or N. Due to issues related to computational convergence, the general applicability as elements, and the complexity arising from the contribution of *f*-electrons to the band structure, lanthanides and actinides have been omitted from our consideration. Based on the host materials in Fig. 1(a–g), respective layered intercalation compounds are shown in Figs. 2–6.

First, Fig. 1(a) shows graphite, a host for the typical layered intercalation compound, Graphite Intercalation Compounds (GICs), with five experimentally reported crystal structures shown in Fig. 2. In Fig. 2 and subsequent figures, “M” represents intercalant element. Symbols G1 ~ G5 are assigned for the five structures to distinguish the structural type, and these five structures are experimentally reported for GICs with some intercalants M. For example, G1 structure was reported in LiC_6_^40^, G2 structure in SrC_6_ and BaC_6_^41^, G3 structure in CaC_6_^42^, G4 structure in CsC_8_^43^ and G5 structure in KC_8_^44^ and RbC_8_^45^. Figure 1(b,c) represent layered materials with octahedral and trigonal prismatic structures, respectively, with compositions AX_2_. The structures of layered intercalation compounds with different coordination and stacking order reported experimentally are shown in Figs. 3, 4, respectively. Figure 1(d–f) show the structures represented by the composition A_2_X_2_ (with the composition ratio of intercalant as 1), exhibiting rock salt, anti-PbO, and PbO structures, respectively. The difference between (e) and (f) is whether the outer layer of the constituent elements is an anion or a cation, determined based on the electronegativity (Allred-Rochow’s scale^46^) of the two elements. The crystal structures of the layered intercalation compounds with these hosts are shown in Fig. 5(a–c). Figure 1(g) shows the layered materials with octahedral structures represented by the composition A_2_Z (Z = C or N), which exhibit termination-free crystal structures of MXene. The crystal structures with this host are shown in Fig. 6, so-called MAX phase crystal structure. For each crystal structure of layered intercalation compounds, symbols are assigned using two characters to distinguish the structural types.

**Fig. 7:**
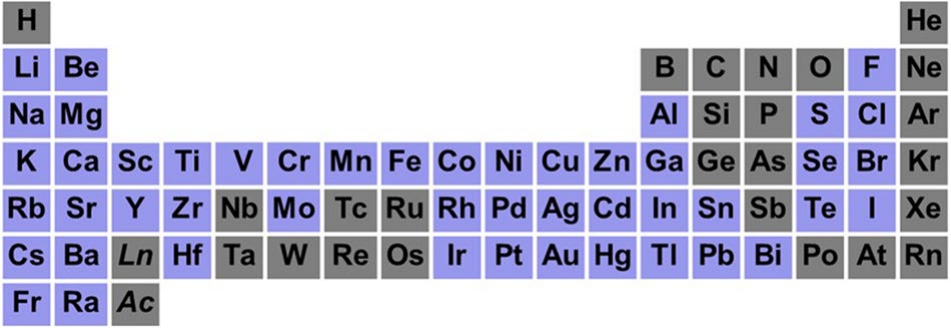
Elements selected as intercalants in the study (shown in blue).

Next, the intercalant elements were selected as shown in Fig. 7. These elements were chosen based on their standard hydration ion Gibbs energy values and ionic radii extracted from the literature^47–51^, excluding lanthanides and actinides, to select ions that are easily ionized and have the potential for intercalation.

**Fig. 8:**
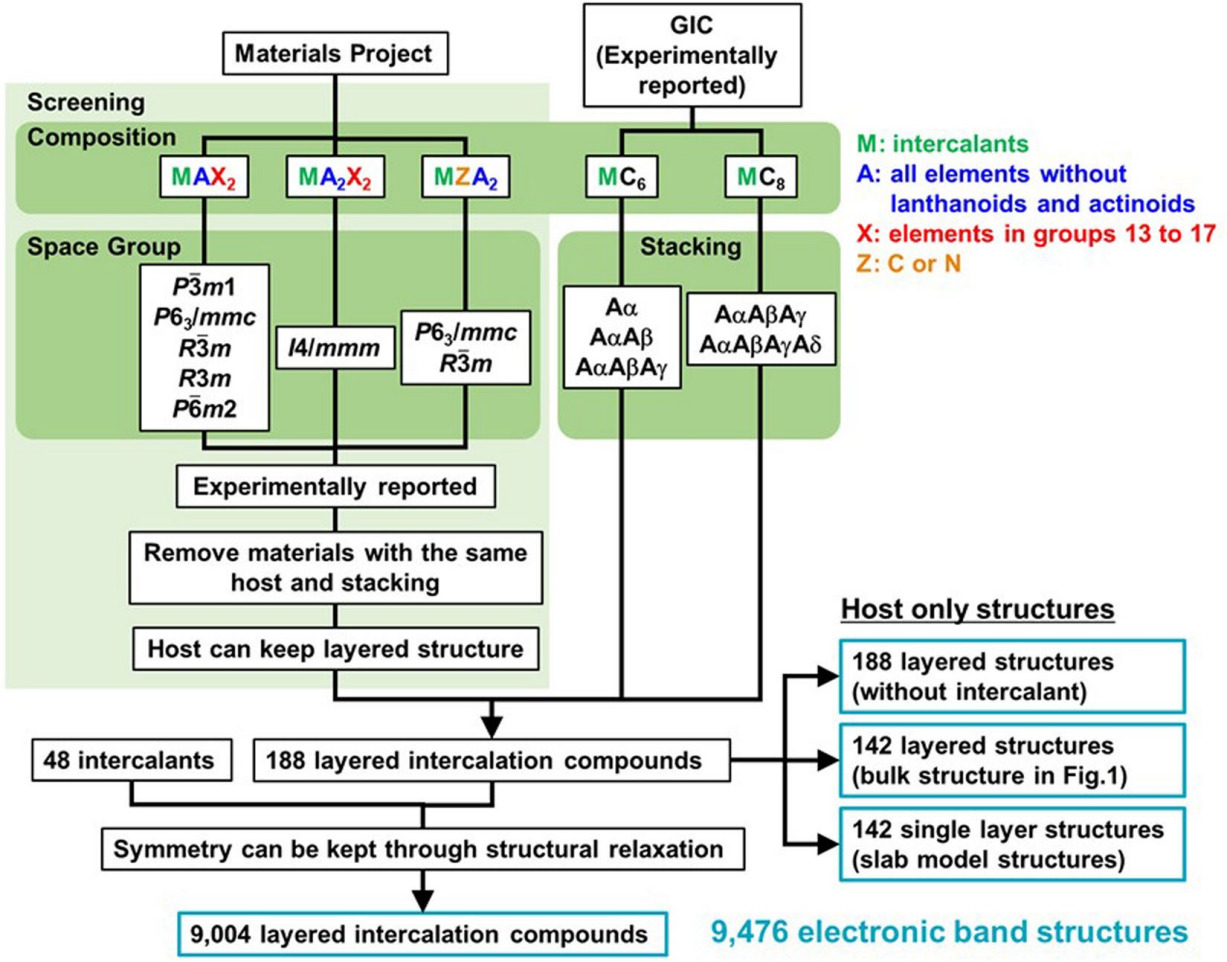
Flowchart illustrating the process of making layered intercalation compounds and host-only layered structures.

The method for making the crystal structures of layered intercalation compounds in this study is illustrated in Fig. 8. Because some GICs with experimentally reported structures are not included in Materials Project^22^, we separated GICs and other layered intercalation compounds. For compounds other than GICs, compounds with crystal structures as shown in Figs. 3–6 were screened based on their composition and space group from Materials Project^22^. Subsequently, only experimentally reported materials were extracted, excluding compounds which both the host and stacking were the same substance. Furthermore, host-only calculations were performed to extract only those layered intercalation compounds for which the host can maintain a layered structure. For GICs, five reported structures as shown in Fig. 2 were simulated. A total of 188 layered intercalation compounds were obtained for these 22 structures. By varying the intercalants among the 48 elements shown in Fig. 7 for these layered intercalation compounds, crystal structures for 9,024 layered intercalation compounds were generated.

Structural relaxations with first-principles calculations were then conducted, and band structure calculations were performed for 9,004 structures of which the space group of crystal structure remained unchanged. Additionally, band structure calculations were conducted for 188 structures with only the host structures obtained by removing the intercalants from the layered intercalation compounds, 142 bulk structures of hosts as shown in Fig. 1, and 142 single-layer slab models of hosts, resulting in a total of 9,472 band structure calculations.

### Conditions of first-principles calculations

Structural relaxations through simulations based on Density Functional Theory (DFT)^52,53^ were performed for screening and structural optimization using the plane-wave basis projector augmented wave method^54^ implemented in the Vienna Ab initio Simulation Package (VASP) code^55–59^. For the simulation of the layered compounds, van der Waals dispersion (vdW) forces have to be implemented to achieve accurate structure and energies^60^. To consider the vdW interaction, we systematically examined various vdW density functional methods^61–65^ for the GICs as the reference, confirming the reproducibility of their crystal structures and the validity of the formation energies. For structural reproducibility, the experimentally reported GICs were calculated using each vdW correction method and the experimentally reported values of the interlayer distances, i.e. 3.67 Å for LiC_6_ (G1-structure)^66^, 4.52 Å for CaC_6_ (G3-structure)^4^, 4.94 Å for SrC6 (G2 structure)^67^, BaC6 (G2 structure) 5.29 Å^68^, KC_8_ (G5 structure) 5.35 Å^69^, RbC_8_ (G5 structure) 5.65 Å^69^, CsC_8_ (G4 structure) 5.94 Å^69^, we verified how much error is introduced to these values. As for the validity of the formation energies, the G1 ~ G5 structures were calculated with the above experimentally reported intercalants Li, Ca, Sr, Ba, K, Rb and Cs, and the formation energies of the experimentally reported structures were compared with the structure with the lowest formation energy in the calculation. The energy difference between the experimentally reported structure and the structure with the lowest formation energy in the calculations was compared. The results are listed in Table 1. Consequently, we selected the rev-vdW-DF2 method proposed by Hamada^64^ because both errors were the lowest. In other words, rev-vdW-DF2 method is superior to the GIC system in terms of structural reproducibility. For the layered intercalation compounds other than GICs, in the system of Cu-intercalated bulk MoS_2_, it has been reported that rev-vdW-DF2 is the best method for vdW correction^70^. Furthermore, in the system of Rb-intercalated bilayer PtTe_2_ (octahedral host structure), it has been reported in a previous study that there is no significant effect on the electronic structure calculations with or without vdW correction by rev-vdW-DF2^71^. Therefore, it seems reasonable to use rev-vdW-DF2 for structural optimization and electronic structure calculations of layered intercalation compounds, with reference to these previous studies.

**Table 1:** Comparison of the MAE of the interlayer distance between the carbon layers (ΔC-C) and the energy difference (Δ*E*, difference between the experimentally reported structure and calculated as the most stable structure) of GICs in each vdW correction method.

vdW correction	ΔC-C	Δ*E* (meV/f.u.)
- (no correction)	1.4%	13.2
vdW-DF2^61^	3.9%	11.1
optPBE^62^	1.2%	8.2
optB86b^63^	1.2%	5.9
rev-vdW-DF2^64^	0.9%	5.9
SCAN + rVV10^65^	1.6%	6.3

The interlayer distances are extracted from refs. ^4,66–69^.

Spin-polarized calculations were performed, but the spin-orbit interaction was not considered. For primitive cell calculations of structural relaxation and electronic structure relaxation, the Brillouin zone (BZ) was sampled by odd-number *k*-point meshes with spacing less than 0.25 Å^−1^, using the Monkhorst-Pack scheme^72^. The electron wave functions were expanded in plane waves up to a kinetic energy cutoff of 650 eV, and the self-consistent field convergence energy was set to 10^−4^ eV. Full structural relaxations of the cell volume and atomic positions were performed until the residual forces on the ions converged to below 0.05 eV/ Å.

After conducting structural relaxation calculations for each primitive cell, optimized structures were fixed and electronic structure relaxation calculations were additionally performed to generate data of electron density distribution of the optimized structure. Subsequently, for the generated electron density data, eigenvalue calculations were performed along high symmetry *k*-paths according to the space group, employing the method proposed by Hinuma *et al*.^73^ and developed in this study. (See below for details.) The eigenvalues were calculated at 40 points between each *k*-point and the band structures were obtained. From the band structure, we extracted the band gap and whether it is a direct or indirect.

### Aligning *k*-path of layered hosts and layered intercalation compounds

The above computational methods have been used to systematically perform first-principles calculations for layered hosts and layered intercalation compounds. Basically, to make *k*-path for electronic band structure, it is appropriate to employ the common approach proposed by Setyawan *et al*.^74^ or Hinuma *et al*.^73^ However, as mentioned above, the *k*-paths may differ between layered hosts and layered intercalation compounds. For the same space group, although the lattice constants should be changed by intercalation, resulting the change of the cartesian coordinates of *k*-points in reciprocal space, comparing *k*-paths before and after intercalation becomes feasible by considering the correspondence of fractional coordinates based on reciprocal lattice vectors. However, comparing the space groups of the host structures shown in Fig. 1 and that of the layered intercalation compounds shown in Figs. 2–6, there are cases where the crystal system or centering type of Bravais lattice changed and BZ also changed. In such cases, the *k*-path in the host and in the layered intercalation compound is not consistent. In general, directly comparing the electronic band structures between crystal structures with different BZs requires employing strategies that may involve increasing computational costs, such as extending the computational cell from a primitive cell to a conventional cell. Therefore, in this study, we developed a novel approach where electronic structure calculations and band structure depictions were conducted for the following five cases, (1–5), by altering the *k*-path while keeping the primitive cell.

**Fig. 9:**
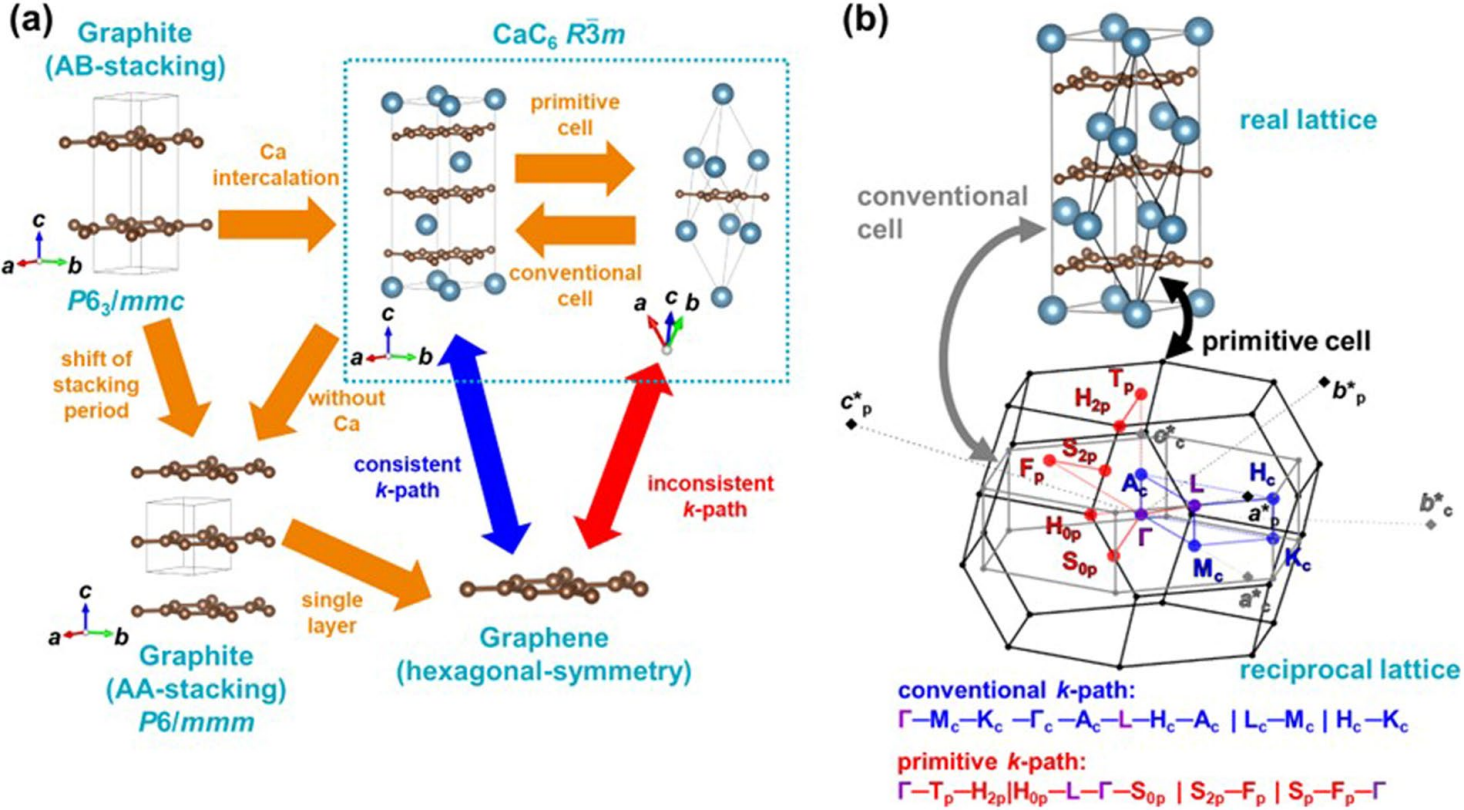
(**a**) Structural relationship between host graphite and Ca intercalated CaC_6_ AαAβAγ-stacking structure. (**b**) Relationship between real lattice, reciprocal lattice and *k*-path of conventional cell and primitive cell in CaC_6_ AαAβAγ. The subscripts of the letters in the *k*-points represent “p” for primitive and “c” for conventional.
  The relationship between the conventional cell (grey line) and primitive cell (black line) in real lattice, BZ in reciprocal lattice, and *k*-path was shown in Fig. 9(b). Graphite with AB-stacking has the space group *P*6_3_/*mmc*, while AA-stacking has *P*6/*mmm*, both are simple hexagonal lattices. In the case of GIC and other hexagonal-host systems, it is preferable to unify the *k*-path taking to simple hexagonal crystals, where hexagonal monolayer stacks without any centring, because their band structures become consistent with the in-plane band structure of graphene or other single layers of hexagonal hosts. However, upon intercalation of Ca, the space group changes to $R\bar{3}m$, acquiring rhombohedral centering. As shown in Fig. 9(b), the band structure aligned with the symmetry of the conventional cell, which has the similar symmetry with the host, cannot be consistent with the *k*-path of the primitive cell. Note that in the dataset of this study, structures with symmetry of *R*3*m* or $R\bar{3}m$ all satisfy the condition of $\sqrt{3}a$ <$\sqrt{2}c$, hence only the setting for this axis length condition of BZ and *k*-path was considered.
  Because the conventional cell of the rhombohedral crystal contains three times as many atoms as the primitive cell, calculating the electronic states of the rhombohedral crystal in its conventional cell and describing the band structures along the *k*-path of the conventional cell to directly compare them with those of the hexagonal host not only leads to increase computational costs but also results in multiple bands overlapping along the same path, making it difficult to extract characteristic features of the band structure. Therefore, in this study, we developed a new method to describe electronic states of the primitive cell using a *k*-path similar to the symmetry of the conventional cell.
  Figure 10 illustrates this approach. First, as shown in Fig. 10(a), by shifting the BZ of conventional cell (grey line) and its *k*-path (blue) by + c*_c_ and -c*_c_, the reciprocal lattice vectors of the conventional cell, and considering two neighboring BZs, the volume of the BZ of conventional cell is tripled. This results in a volume equivalent to that of the BZ of primitive cell (black line). At this point, the first conventional cell’s subscripts of *k*-points (shown in blue) are denoted as c (referred to as c-cell), the subscripts of *k*-points for the cell shifted by -c*_c_ (shown in green) are denoted as c1 (c1-cell), and those shifted by + c*_c_ (shown in orange) are denoted as c2 (c2-cell). Furthermore, to further distinguish them from the original *k*-points, the ‘prime’ was used. Thus, 18 *k*-points and 27 *k*-paths (9 each for blue, orange, and green) are generated as shown in Fig. 10(a). While these *k*-paths are (partially) inequivalent in the primitive cell, considering symmetry operations of the crystal structure (*R*3*m*, $R\bar{3}m$) such as three-fold rotation axis and mirror, shifts by the unit reciprocal lattice vectors of the BZ of primitive cell (a*_p_, b*_p_, c*_p_), and inverse symmetry of *k*-space under time-reversal symmetry (*ε*(-***k***) = *ε*(***k***)), equivalent points can be identified, indicating the existence of equivalent paths. Therefore, it suffices to calculate the minimum paths.

1. From hexagonal host to rhombohedral layered intercalation compounds
  When the host has a hexagonal crystal structure, there are cases where the space group of layered intercalation compounds has the rhombohedral centering (*R*3*m*, $R\bar{3}m$) depending on the arrangement of intercalants and stacking order, such as the change from graphite to G3 structure or from octahedral host to O4 ~ O6 structures. As an example, the change from graphite to CaC_6_ (G3 structure) is shown in Fig. 9(a), showing the relationship between the structures of the host and layered intercalation compound.

**Fig. 10:**
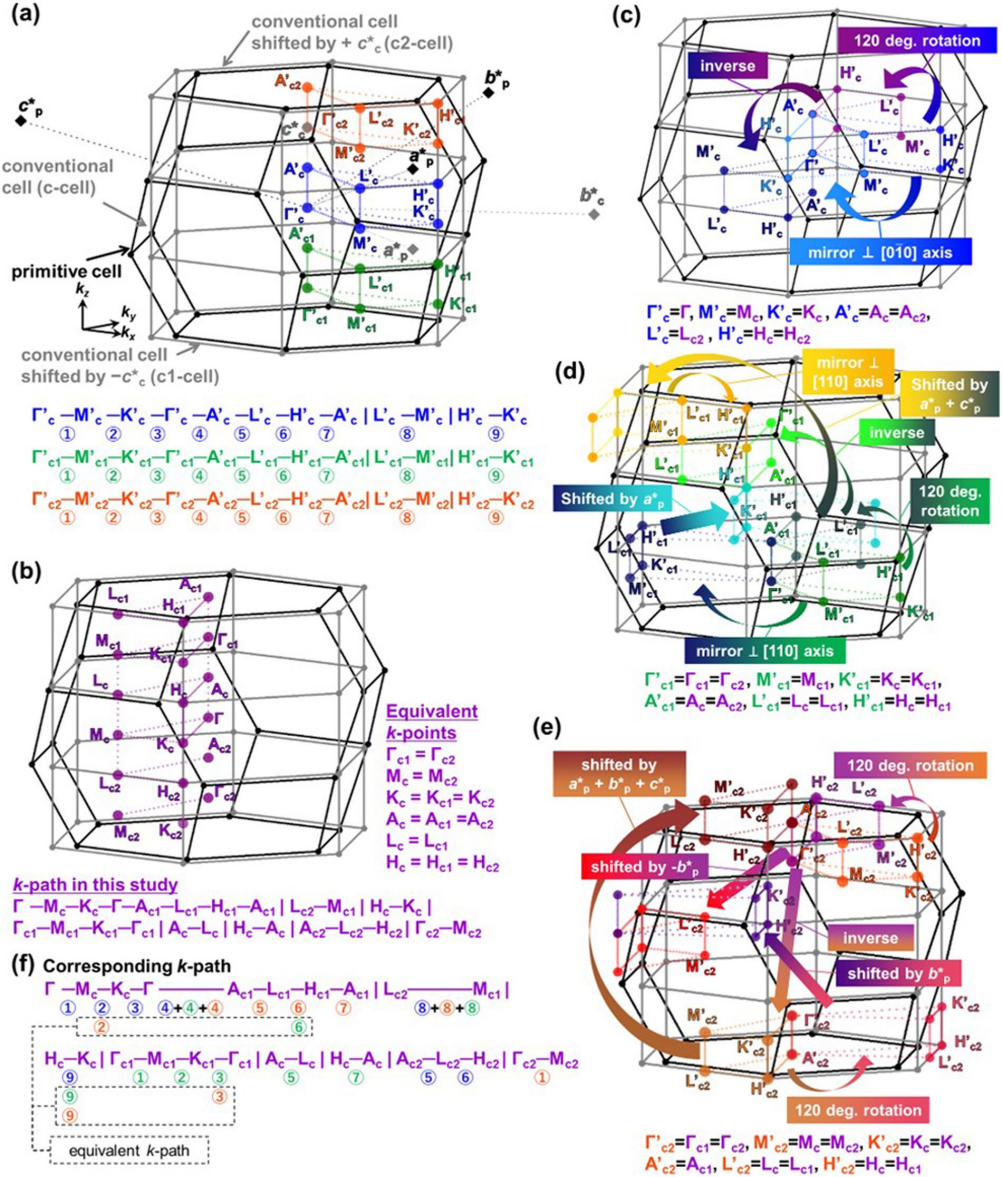
Construction of the conventional-like *k*-path within the primitive BZ of *R*3*m* and $R\bar{3}m$ in this study. (**a**) BZs of the primitive cell, conventional cell (c-cell), and conventional cells shifted by -c*_c_ (c1-cell) and + c*_c_ (c2-cell), and the corresponding *k*-paths for each conventional cell. (**b**) Equivalent *k*-points and *k*-path for the BZ of primitive cell with conventional-like symmetry developed in this study. (**c**) Conversion of the *k*-points in the c-cell. (**d**) Conversion of the *k*-points in the c1-cell. (**e**) Conversion of the *k*-points in the c2-cell. (f) Correspondence between the *k*-path in the three conventional cells and that was developed in this study.
  Figure 10(b) presents the minimal *k*-path obtained in this study, which can reproduce the *k*-path of conventional cell without band overlap. Additionally, Fig. 10(c,d) illustrate the symmetry operations applied to the c-cell, c1-cell, and c2-cell, indicating which points on the *k*-path in this study coincide with them. For instance, Fig. 10(c) demonstrates the symmetry operations for the c-cell, where it is evident that Γ’_c_ = Γ_c_ and A’_c_ = A_c_, while H’_c_ = H_c_ and K’_c_ = K_c1_ are deduced due to the mirror symmetry elements about mirror planes perpendicular to [$0\bar{1}0$] axis in the real lattice. Furthermore, after a 120-degree rotation around the ***c***-axis in real space followed by an inversion operation (-***k**** = ****k***), it is found that M’_c_ = M_c_, K’_c_ = K_c_, A’_c_ = Ac_2_, L’_c_ = L_c2_, and H’_c_ = H_c2_. This enables the identification of equivalent points and paths in the primitive cell, allowing for the generation of a minimal *k*-path while preserving the symmetry of the conventional cell. Figure 10(d,e) also describe the operations necessary to derive equivalent *k*-points in c1-cell and c2-cell.
  We compared the electronic band structures of CaC_6_ AαAβAγ-stacking ($R\bar{3}m$) computed using the developed *k*-path in this study with that obtained using a general method, in Fig. 11. All band structures are colored by the contribution ratio of Ca. Figure 11(a) shows the band structure described using the developed path for the primitive cell in this study. Figure 11(b) shows the overlayed band structure of (a) with the same symbol and constructed the band structure for the conventional cell. Figure 11(c) shows the band structure described in the *k*-path of conventional cell from the conventional cell calculation for the comparison. Figure 11(d) shows the band structure described in the *k*-path of primitive cell from the primitive cell calculation. Figure 11(e) compares the *k*-path in BZ. As evident from the figure, Fig. 11(b) coincides with Fig. 11(c), demonstrating that band structures computed for the primitive cell can reproduce those for the conventional cell calculation.

**Fig. 11:**
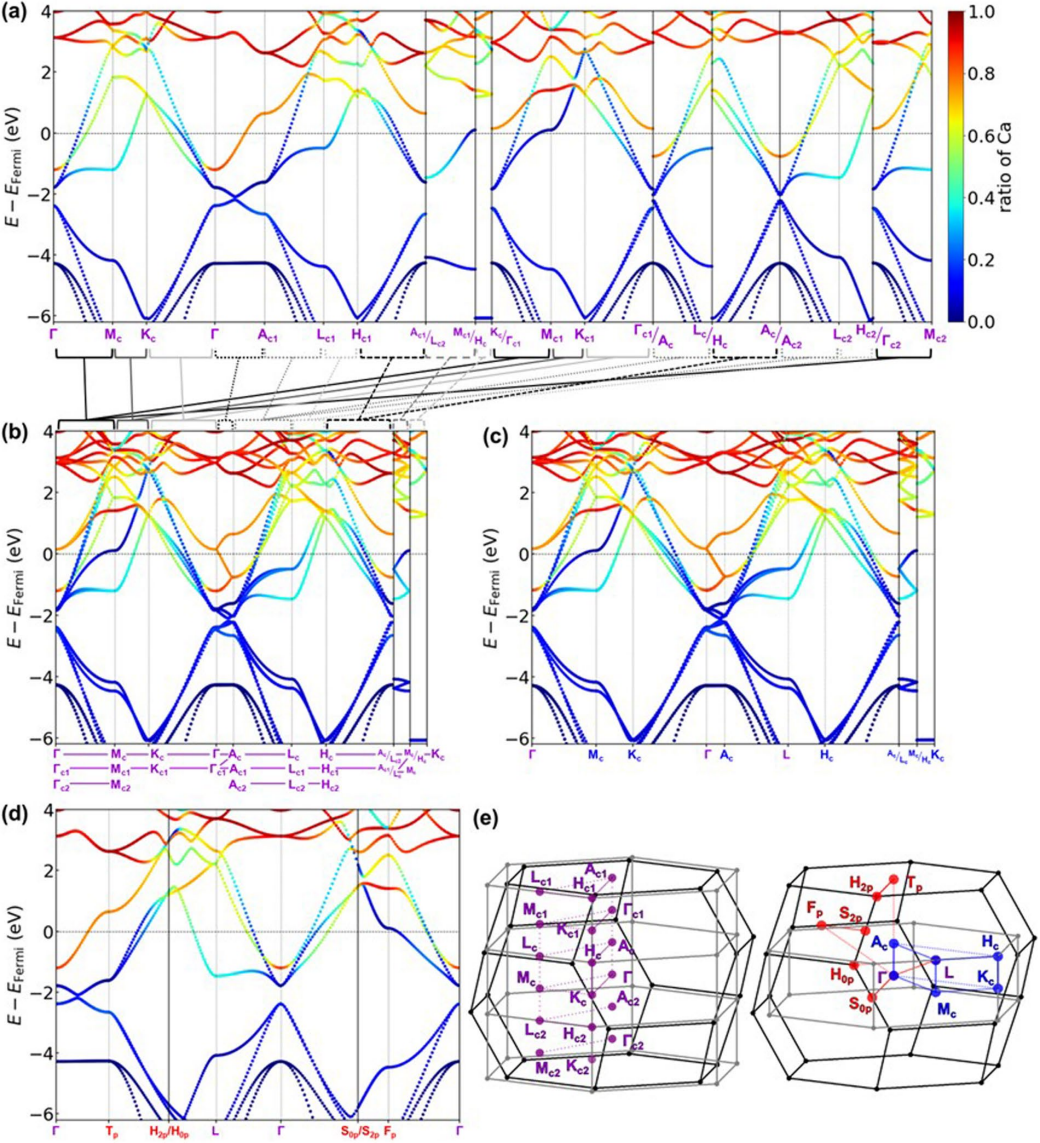
Band structures of CaC_6_ AαAβAγ-stacking structure. (**a**) Band structure computed for the primitive cell and the developed *k*-path in this study. (b) Constructed conventional band structure from primitive cell calculation and developed *k*-path as shown in (**a**). The corresponding *k*-path is shown at the bottom. (**c**) Band structure obtained from conventional cell calculation and the *k*-path of conventional cell. (**d**) Band structure computed for the primitive cell and the *k*-path of primitive cell. (**e**) Comparison of *k*-paths in reciprocal lattice.
  This method allows for the description of structures that maintain host symmetry while minimizing band overlap, facilitating the extraction of band characteristics. For instance, in Fig. 11(a), the band structure of Γ-A_c1_ intersects the Fermi energy, representing the free-electron-like inter layer band, which is associated with the superconducting properties^37–39^. However, as shown in Fig. 11(c), band folding occurs in the conventional cell calculation, making extraction of the feature of band structure challenging. Additionally, it was found that the electronic band structure of the primitive cell in Fig. 11(d) does not provide band structures along high-symmetry *k*-paths comparable to those within the plane and between planes of the host structure. This highlights the suitability of the *k*-path devised in this study, as shown in Fig. 11(a), for describing the band structure.
  One point should be noted: the *k*-paths employed in this study have the Γ-L and Γ-F_p_ of the rhombohedral *k*-paths omitted, as can be seen by comparing Fig. 11(a–e). These two paths connect the Γ point of the rhombohedral BZ to points L and F_p_, which are the centers of the BZ faces, but they have been omitted because it is considered that paths parallel or perpendicular to the layers are important in layered intercalation compounds. In fact, this path has not been calculated in several previous studies of the band structure of rhombohedral layered intercalation compounds^75–77^, and it was decided to omit it in the present study because the main focus is on the correspondence between the paths of the host and the interlayer compounds. On the other hand, it is possible that some important electronic states may be missed from the perspective of the comparison of host and interlayer compounds, which is the main focus of this study. A recent study^78^ have reported that even the commonly used *k*-path method of Setyawan *et al*.^74^ and Hinuma *et al*.^73^ is not sufficient, and it is controversial what a sufficient *k*-path should look like. In this study, in order to complement the *k*-path of the Hinuma *et al*. method, which was omitted from our developed *k*-path, we have also calculated the basic *k*-path for primitive cells separately and included them in the database.
2. From graphite to MC_8_ A*α*A*β*A*γ*A*δ*-stacking structure (G5)
  In the experimentally reported GICs, KC_8_^44^ and RbC_8_^45^ have the structure which is hard to compare *k*-path directly with host graphite. In this structure of GIC A*α*A*β*A*γ*A*δ*-stacking (G5), the space group *Fddd*, face-centered orthorhombic, lacks 3-fold or 6-fold rotational symmetry, making it incompatible with the *k*-path of hexagonal graphite. In some previous studies^79–81^, the electronic structure was calculated as orthorhombic and discussed in a path close to the hexagonal symmetry of graphite, which does not facilitate comparison of the host and layered intercalation compounds. Therefore, in this study, we aligned the graphite side as an orthorhombic-like cell to compute the band structure, facilitating comparison of band structures between the host and the layered intercalation compound. Furthermore, due to the face-centered lattice centering, the band structure of the primitive cell becomes incompatible with the orthorhombic cell of the conventional cell, requiring a similar refinement in the arrangement of the *k*-path as in case (1). Figure 12(a) shows the relationship between the orthorhombic cell of graphite adopted in this study and the crystal structure of K-intercalated Graphite (KC_8_ A*α*A*β*A*γ*A*δ*-stacking), and Fig. 12(b) shows the relationship between the conventional cell and primitive cell in real space and the BZ, as well as the *k*-path in reciprocal space. It is noted that in this study, structures with the *Fddd* space group all satisfy the condition *a*^*−*2^ > *b*^−2^ + *c*^−2^, thus considering only those *k*-paths and BZs satisfying this axis length condition.

**Fig. 12:**
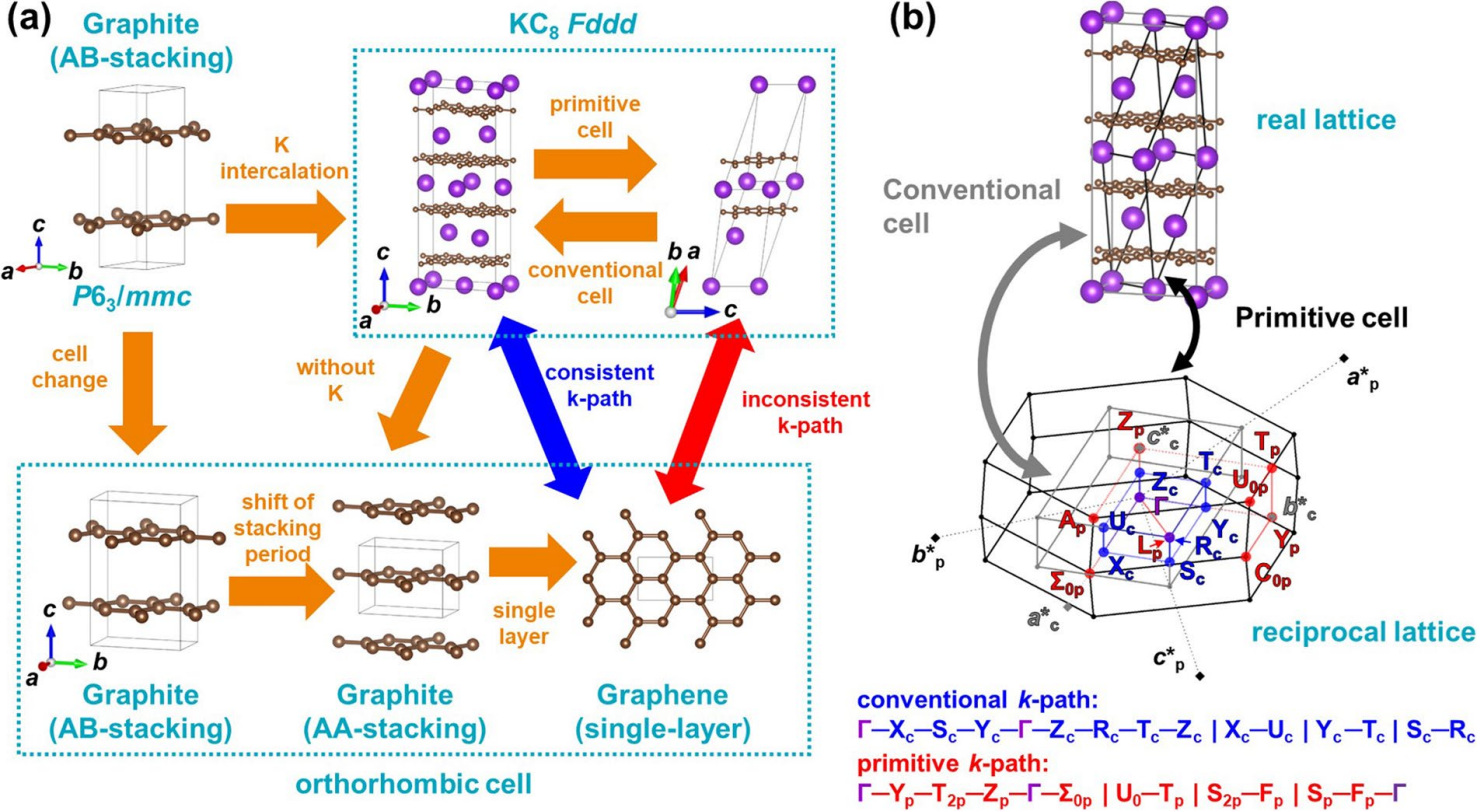
(**a**) Structural relationship between host graphite and K-intercalated KC_8_ AαAβAγA*δ*-stacking structure. (**b**) Relationship between the real lattice, BZ and *k*-path of the conventional cell and primitive cell of KC_8_ AαAβAγA*δ*-stacking. The subscripts of the letters in the *k*-points represent “p” for primitive and “c” for conventional.
  In Fig. 13, the method for aligning the *k*-paths of the layered host with primitive orthorhombic cell and layered intercalation compound with face centered orthorhombic cell is shown, similar to (1). Firstly, by shifting the BZ (grey line) and *k*-points (shown in blue) of the conventional cell by -c*_c_, -b*_c_, and -b*_c_-c*_c_, three neighboring BZs are considered, resulting in quadrupling the volume of the conventional cell’s BZ. This volume matches that of the BZ of primitive cell. At this point, the initial conventional cell’s subscripts of *k*-points are denoted as c (referred to as the c-cell), that for the cell shifted by -c*_c_ (shown in orange) as c1 (c1-cell), that for the cell shifted by -b*_c_ (shown in green) as c2 (c2-cell), and that for the cell shifted by -b*_c_-c*_c_ (shown in magenta) as c3 (c3-cell). To further distinguish them from original *k*-points, the primes were used. Thus, 32 *k*-points and 48 *k*-paths are generated, as shown in Fig. 13(a). These *k*-paths are (partially) inequivalent in the primitive cell, and similar to (1), by considering symmetry operations of the crystal structure (*Fddd*), such as 2-fold rotation axes around [100], [010], [001], shifts by the unit reciprocal lattice vectors of the BZ of primitive cell (a*_p_, b*_p_, c*_p_), and inverse symmetry of *k*-space assuming time-reversal symmetry (*ε*(-***k***) = *ε*(***k***)), equivalent points can be identified to determine the minimal path. This results in the developed *k*-path in this study and its equivalent *k*-points, as shown in Fig. 13(b). Furthermore, Fig. 13(c–f) show the symmetry operations applied to the c, c1, c2, and c3-cells and show which points on the *k*-path in this study correspond to them. The c-cell corresponds without symmetry operations, while the other three cells show equivalent points induced by symmetry operations. Although the details of symmetry operation are omitted because they are huge, as shown in Fig. 13(g), all *k*-paths in Fig. 13(a) correspond to those in this study.

**Fig. 13:**
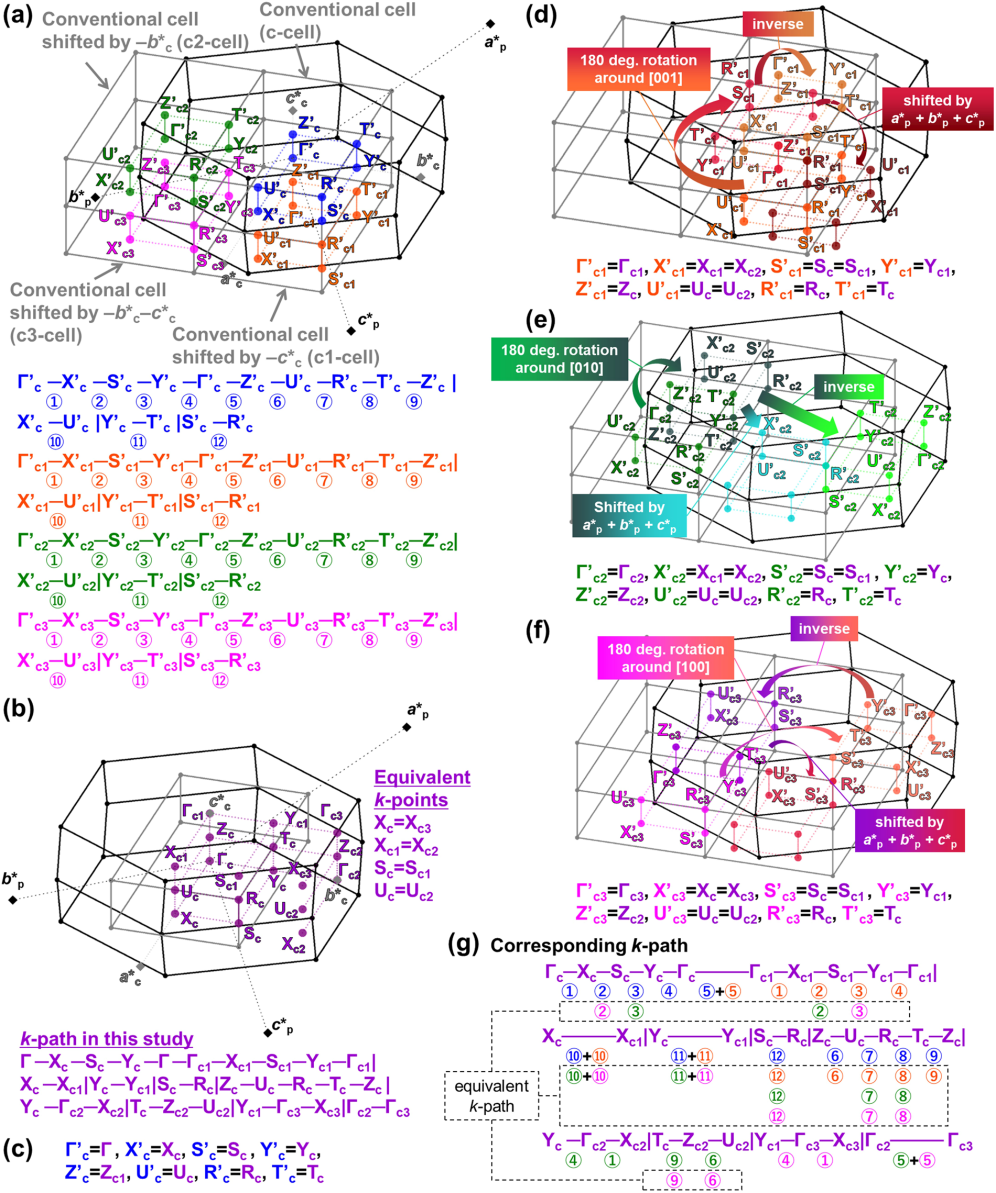
Construction of conventional-like *k*-path in the primitive BZ of *Fddd* in this study. (**a**) BZs of the primitive cell, conventional cell (c-cell) and conventional cells shifted by -c*_c_ (c1-cell), -b*_c_ (c2-cell), and -b*_c_-c*_c_ (c3-cell), and the corresponding *k*-path for each conventional cell. (**b**) Equivalent *k*-points and *k*-path for the BZ of the primitive cell with conventional-like symmetry developed in this study. (**c**) Corresponding *k*-points in the c-cell. (**d**) Conversion of *k*-points in the c1-cell. (**e**) Conversion of *k*-points in the c2-cell. (**f**) Corresponding *k*-path in the four conventional cells.
3. From tetragonal host to body-centered tetragonal layered intercalation compounds
  In the case where the host has primitive tetragonal lattice (Anti-PbO or PbO in Fig. 1) and the intercalant insertion induces an in-plane-half-unit cell shift in the host, the space group with a body-centered lattice (*I*4/*mmm*) is obtained. Taking the compound CaFe_2_As_2_ as an example, Fig. 14(a) shows the relationship between the host and the layered intercalation compound under this condition, and (b) shows the relationship between the real lattice, BZ and *k*-path of the conventional cell and primitive cell. It is noteworthy that the host material labeled as FeAs in this figure is not a reported synthesized compound. Similar to (1, 2), it is evident that the band structure obtained from the *k*-path of the primitive cell aligned with the space group does not match the symmetry of the host. Note that in this study, structures with the *I*4/*mmm* space group all satisfy the condition *c* > *a*, hence, the BZ and *k*-path are considered only under this axis length condition.

**Fig. 14:**
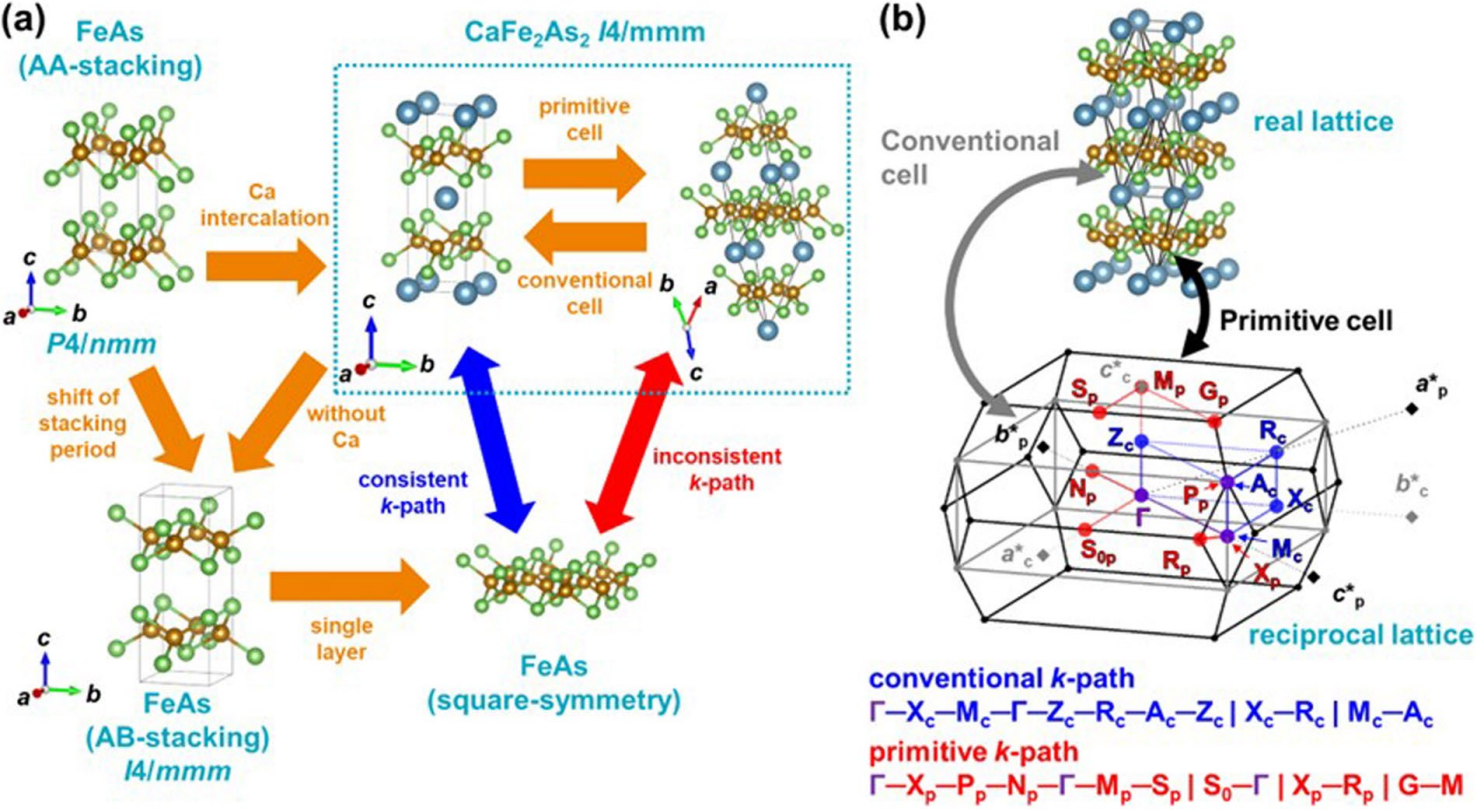
(**a**) Structural relationship between the host FeAs and Ca intercalated CaFe_2_As_2_. (**b**) Relationship between the real lattice, BZ, and *k*-path of the conventional cell and primitive cell in CaFe_2_As_2_. The subscripts of the letters in the *k*-points represent “p” for primitive and “c” for conventional.
  Figure 15 shows the *k*-path with conventional-like symmetry developed in this study for the primitive BZ, following similar methodologies as in (1, 2). Firstly, we consider neighboring BZ of conventional cell (grey line) and that of *k*-points (shown in blue) by shifting the reciprocal lattice unit vector of the conventional cell -c*_c_. This doubles the volume of the BZ, equivalent to the first BZ of the primitive cell. At this point, the first conventional cell’s subscripts of *k*-points (shown in blue) are denoted as c (referred to as c-cell), the subscripts of *k*-points for the cell shifted by -c*_c_ (shown in green) are denoted as c1 (c1-cell). Thus 12 *k*-points and 18 *k*-paths are generated, as described in Fig. 15(a). This *k*-path is partially non-equivalent in the primitive cell, and similar to above crystal systems in (1, 2), considering symmetry operations of the crystal structure (mirror planes) and shifts by the unit reciprocal lattice vector of the primitive cell’s a*_p_, enables the determination of equivalent points, allowing for the identification of the minimal path. We illustrate the resulting *k*-path developed in this study along with the equivalent *k*-points in Fig. 15(b), and the operations applied to the c1-cell to derive this path in Fig. 15(c). Finally, Fig. 15(d) demonstrates the corresponding *k*-path between (a) and (b), indicating that all *k*-paths in (a) are appropriately matched with those developed in this study.

**Fig. 15:**
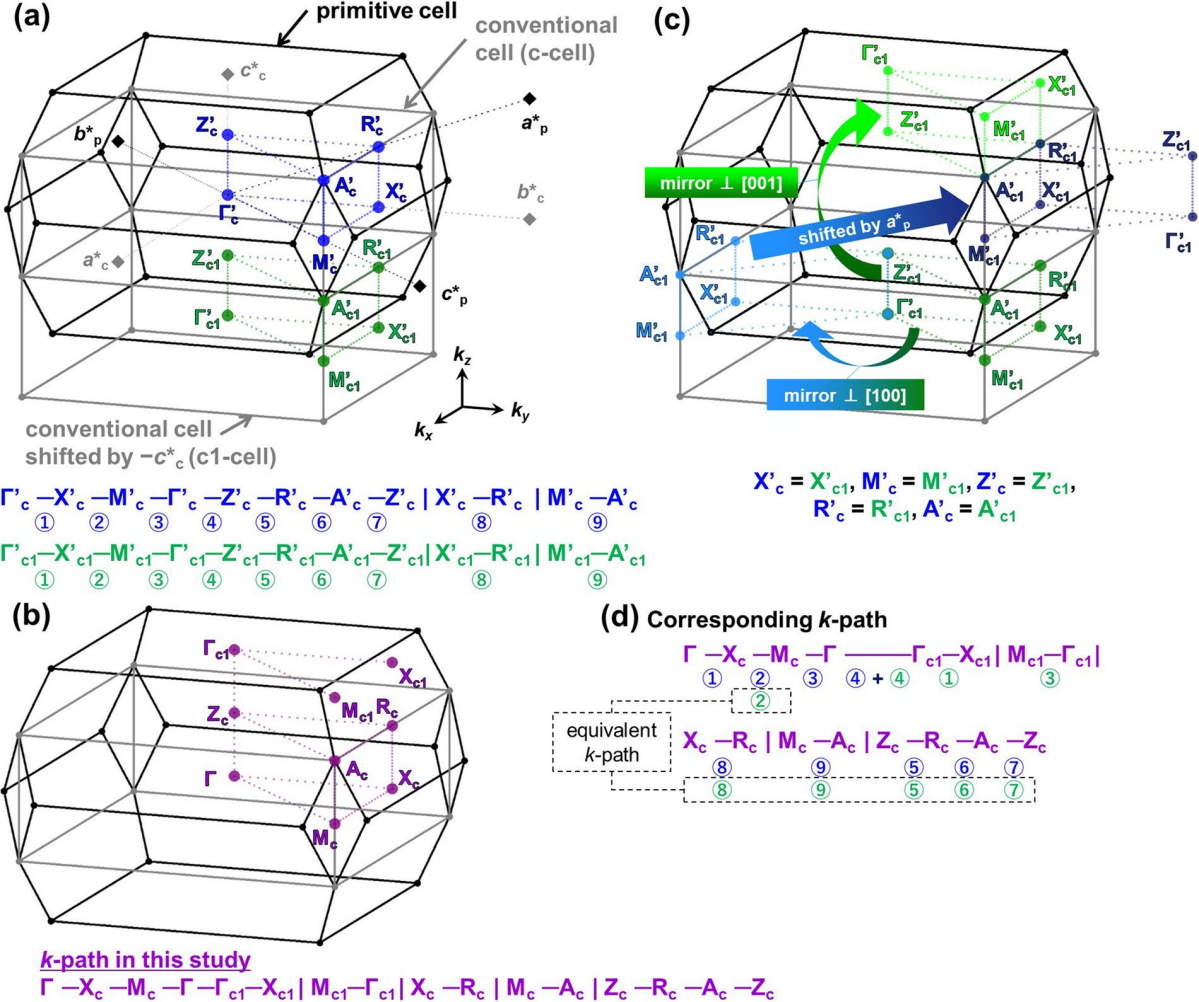
Construction of the conventional-like *k*-path in the primitive BZ of *I*4/*mmm* in this study. (**a**) BZs of the primitive cell, conventional cell (c-cell), and conventional cell shifted by -c*_c_ (c1-cell), along with the *k*-path for each of the conventional cells. (**b**) *k*-path for the primitive cell Brillouin zone with conventional-like symmetry developed in this study. (**c**) Conversion of the *k*-path in c1-cell and equivalent *k*-points in c-cell and c1-cell. (**d**) Correspondence of the *k*-path in two conventional cells and this study.
4. Host having the rock salt structure (space group ${Fm}\bar{3}m$)
  In this case, the band structure of the bulk host based on the space group ${Fm}\bar{3}m$ is incompatible with the tetragonal symmetry of the single layer host. Therefore, in this study, as shown in the example of NiRb_2_O_2_ in Fig. 16, we interpreted the rock-salt structure as the AA-stacking of the layered host and calculated the band structures of AA-stacking and AB-stacking using a tetragonal-like cell. Additionally, since the space group of the layered intercalation compound is *I*4/*mmm*, the band structures were computed using the method described in (3). It is noteworthy that the material referred to as RbO with a rock-salt structure in this figure is not an experimentally reported structure; however, it is virtually calculated as the host structure for database.

**Fig. 16:**
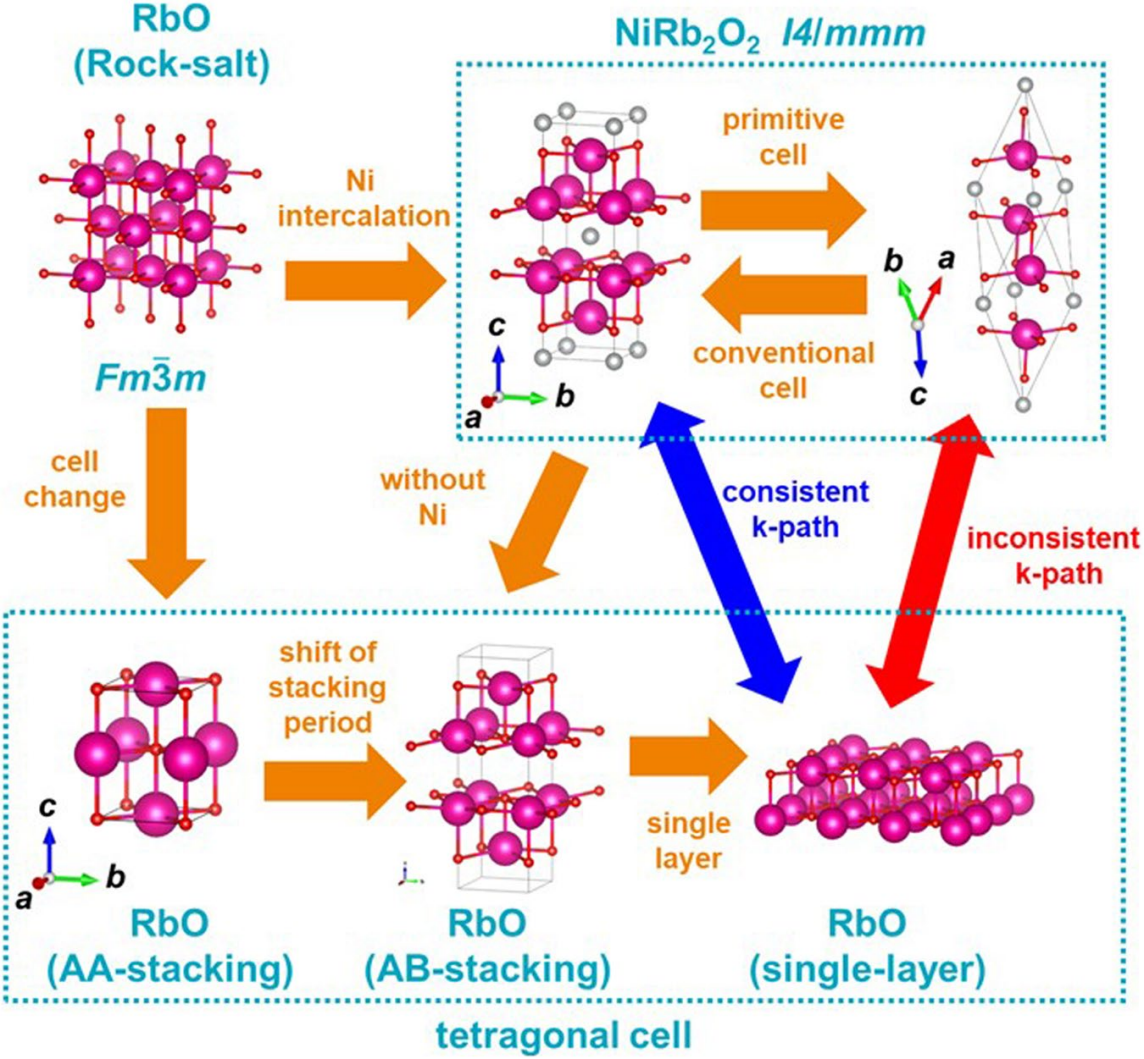
Structural relationship between the host RbO and Ni intercalated NiRb_2_O_2_, and the calculated cell as the host with tetragonal-like symmetry in this study.
  It is also noteworthy that AA-stacking of rock-salt structure is not crystallographically conventional. However, the purpose of this study is comparing the band structure of the host and layered intercalation compounds and trace the change of band structure by intercalation. Therefore, to compare the host and layered intercalation compounds, we propose new method as shown in Fig. 16. In addition, as described above in (1), the band structures of rock salt structure calculated with the basic *k*-path of Hinuma *et al*.^73^ are also separately added to the database.
5. Symmetry of the layered intercalation compound is increased due to “self-intercalation”

**Fig. 17:**
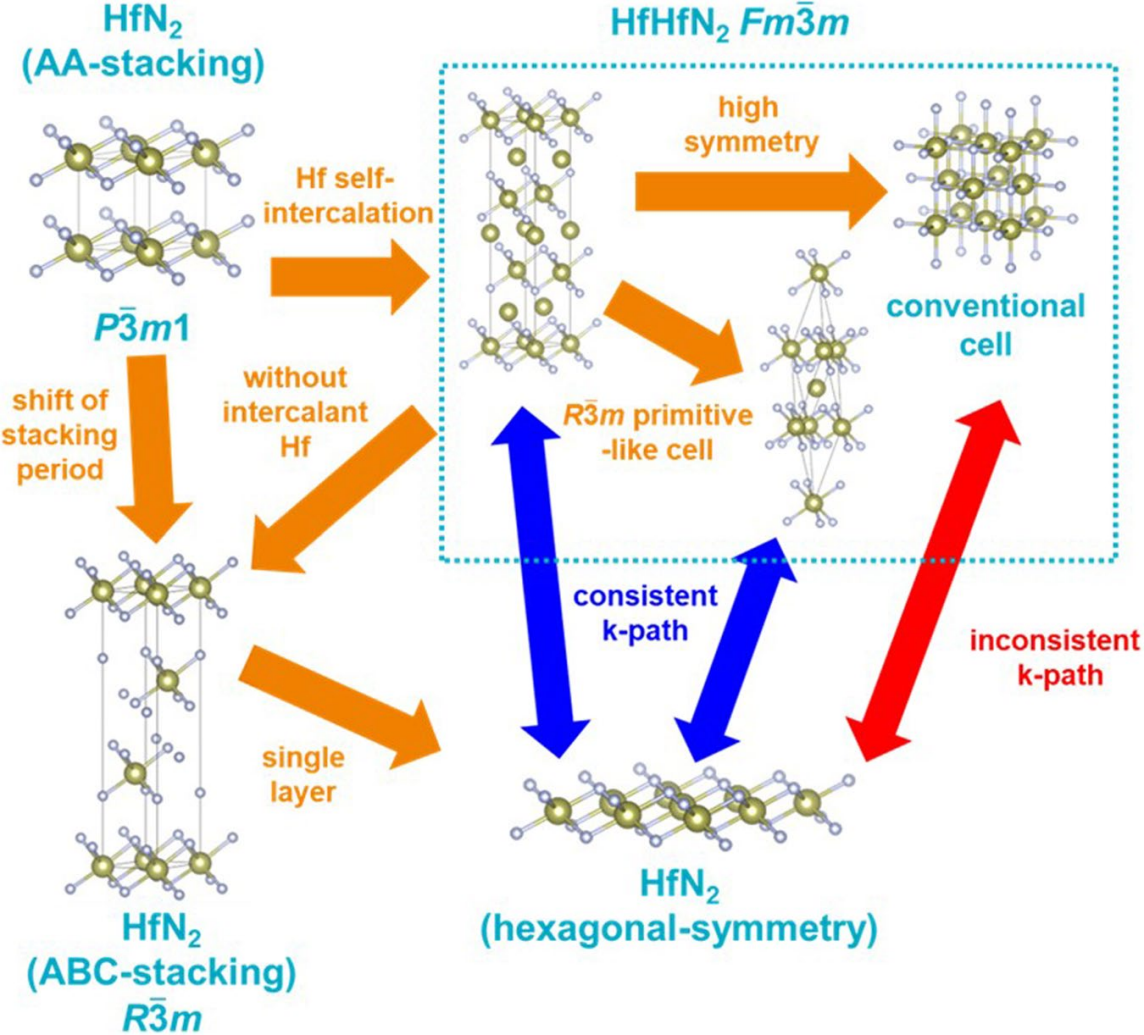
Structural relationship between host HfN_2_ and Hf intercalated HfHfN_2_, and calculated cell as layered intercalation compounds with $R\bar{3}m$ primitive-like cell in this study.

The word “self-intercalation” means the intercalant atom is native to host materials. For example, in our calculation, when Sr is regarded as an intercalant in SrHfO_2_ (O3-structure) and Sr is replaced by Hf, the symmetry of the layered intercalation compound is enhanced with structural optimization and the space group changes from $R\bar{3}m$ to ${Fm}\bar{3}m$. The phenomenon where the same element as those composing the host are intercalated is referred to as “self-intercalation” and has been reported in many previous studies^13,82–88^. For such self-intercalation with high symmetry systems, electronic structure calculations were performed considering the primitive cell structure of $R\bar{3}m$ from the ${Fm}\bar{3}m$ structure as shown in Fig. 17 and the band structure was described using the *k*-path for rhombohedral described in (1).

### Type of structure in which band structure is calculated

**Fig. 18:**
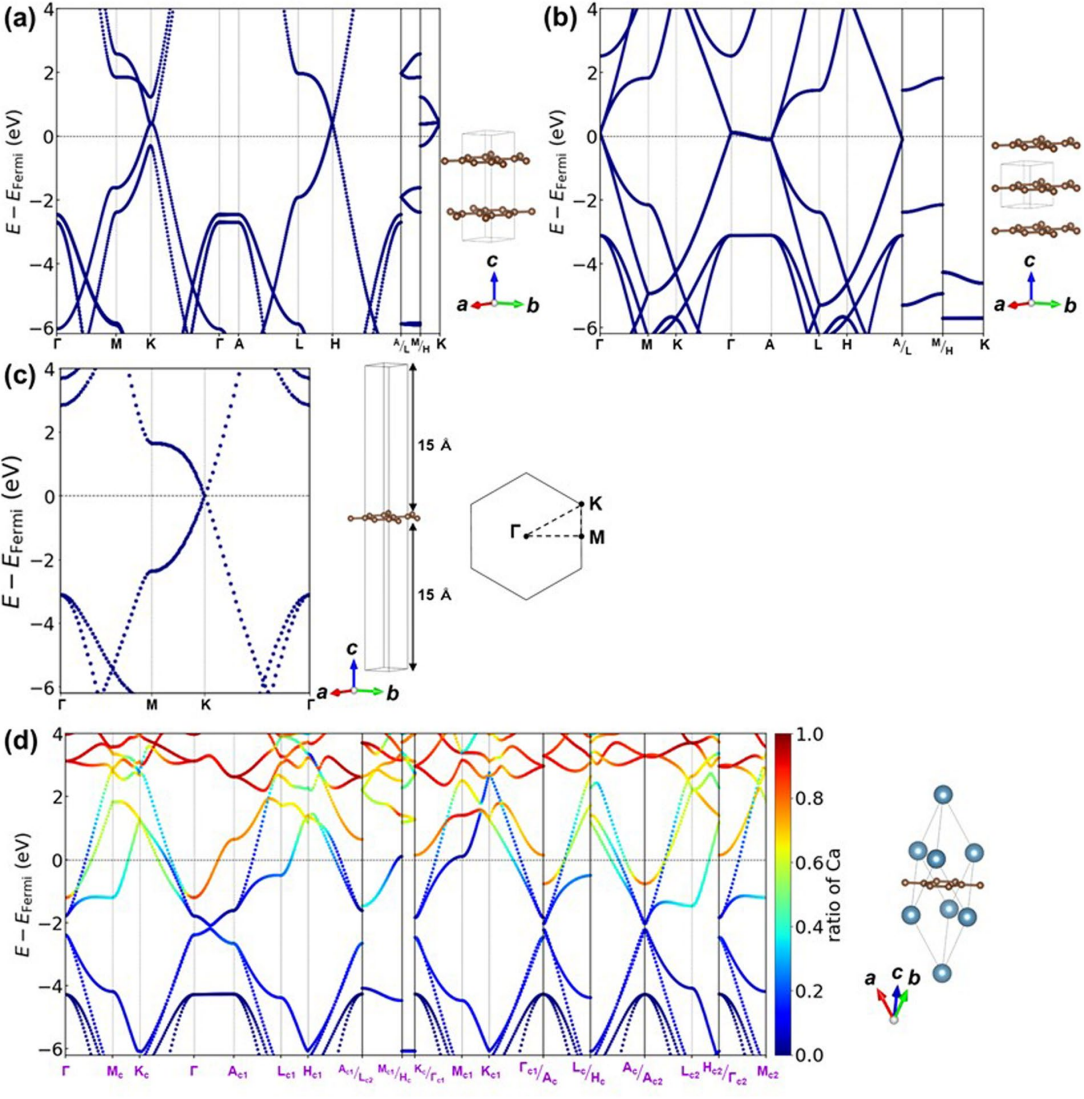
Band structures of (**a**) Graphite host (AB-stacking bulk structure). (**b**) Graphite host (AA-stacking structure, Ca excluded structure from CaC_6_). (**c**) Graphene (slab-model). (**d**) The primitive cell of CaC_6_-AαAβAγ stacking structure with *k*-path developed in this study.

As shown in the flowchart of Fig. 8, electronic structure calculations were performed on a total of 9,004 layered compound crystal structures, and three types of host only structures; (i) 188 layered structures without intercalant (the structures from which only intercalant was removed), (ii) 142 layered bulk structures as shown in Fig. 1 and (iii) 142 single layer structure in slab models, resulting in a total of 9,472 structures. For structures corresponding to the cases described in above (1–5), band structure calculations were carried out along the *k*-points path as described for each part. Examples of the band structures are shown in Fig. 18, using a typical GIC, CaC_6_. Figure 18(a) shows the band structure of bulk graphite as the host, (b) shows the band structure when only Ca is removed from CaC_6_, (c) shows the band structure of graphene as a slab model, and (d) shows the band structure calculated using the *k*-path obtained from the primitive cell of CaC_6_ by using the method described in Fig. 10. As evident from the Fig. 11, the band structure obtained using this method, which creates and visualizes paths consistent with the host structure, facilitates the extraction of features, particularly concerning how the band structure changes from the structure without Ca (Fig. 18(b)) to that with Ca (Fig. 18(d)). Thus, it can be said that a useful database has been established for analyzing band structure variations induced by intercalants.

### Estimation of intercalation energy and formation energy

The inte-rcalation energy *E*_int_ was calculated as the energy required for intercalation using the following Eq. (1),

1 $${E}_{\mathrm{int}}={E}_{{\rm{tot}}}\left(\text{M} \mbox{-} \text{Host}\right)-{E}_{{\rm{tot}}}\left(\text{M}\right)-{E}_{{\rm{tot}}}\left(\text{Host}\right)$$

where *E*_tot_(M-Host), *E*_tot_(M) and *E*_tot_(Host) are the calculated total energies of the layered intercalation compound, the standard state of the intercalant, and the host, respectively. The unit of *E*_int_ is eV/formula unit (f.u.). *E*_int_ can be interpreted as the reaction energy between the intercalant and the host, allowing for a discussion of the stability of the interlayer compound.

While a negative *E*_int_ does not imply that the reaction proceeds because the host is often a virtual structure, all hosts in our constructed database have been experimentally reported as a part of stable layered intercalation compounds with one or more intercalants. Therefore, if their *E*_int_ values are lower than those of the experimentally reported compounds, they could potentially be synthesized through topochemical reactions like ion exchange. Additionally, by replacing *E*_tot_(Host) with Σ*E*_tot_(Host-elements) in Eq. (1), where Σ*E*_tot_(Host-elements) represents the sum of total energy of the standard state of element constructing the host, the formation energy (*E*_f_) of the layered intercalation compounds were calculated. A negative *E*_f_ value suggests that the reaction may proceed from the standard state of elements.

## Data Records

The list including compositions, host and intercalant information, energy-related data (Fermi energy, *E*_int_, *E*_f_) for 9,024 layered intercalation compounds (LC), and physical properties derived from band structures (presence of spin splitting, band gap (up and down), whether the band gap is direct or indirect) for 9,004 of them is available on figshare^89^ under the file name “LC-database.xlsx.” Additionally, for the hosts, similar data are provided for 142 layered host bulk (LHB) crystal structures as described in Fig. 1 in “LHB-database.xlsx”, the intercalant removed structures of 188 mother substances obtained through screening in Fig. 8, in other words, the structures of the layered host with intercalant vacancies (LHV) in “LHV-database.xlsx”, and 142 single-layer structures of layered hosts created using slab models (LHS) in “LHS-database.xlsx”.

### Raw VASP files

Input and output files of VASP, excluding PAW potentials, are available on figshare^89^. Corresponding to the above list files, necessary input files (“INCAR”, “KPOINTS” (line-mode type, “KPOINTS-mesh” (Monkhorst-Pack^72^ type) and “POSCAR”) and output files (“vasprun.xml”) for reproducing the calculations are provided in folders named “LC-database”, “LHB-database”, “LHV-database”, and “LHS-database”. All of these databases contain the results of band structure calculations employing the *k*-path developed in this study as described in “Methods” part. Additionally, for comparison, the band structures of layered intercalation compounds have been calculated using the basic *k*-path of Hinuma *et al*.^73^ are provided for, if the structure of LC is “G3”, “G5”, “O4”, “O6”, “T6”, “RS”, “AP” or “PO” in the “LC-primitive-database”. Then the band structure of the basic *k*-path of the host with rock-salt structure in the host bulk is included in the folder “LHB-primitive-database”. These files are compressed in “tar.gz” format. Band structures can be extracted from “vasprun.xml.” However, due to the low reliability of Fermi energy obtained from band structures, when plotting, it is necessary to use the Fermi energy listed in the “.xlsx” files for each material group. Additionally, information about the VASP PAW potentials, including which potential was used for each element, is recorded in the “Potential.xlsx” file.

### Band structure data visualization script

A python script file (“plot_bands.py”) is available in figshare^89^ that can be used to plot the band structure of a material specified by the number listed in the .xlsx file. As shown in above figures, the script can color the projection of specific element and orbital. Instructions for the required environment and variable settings within the files are provided in readme.txt.

## Technical Validation

### Calculation conditions

**Fig. 19:**
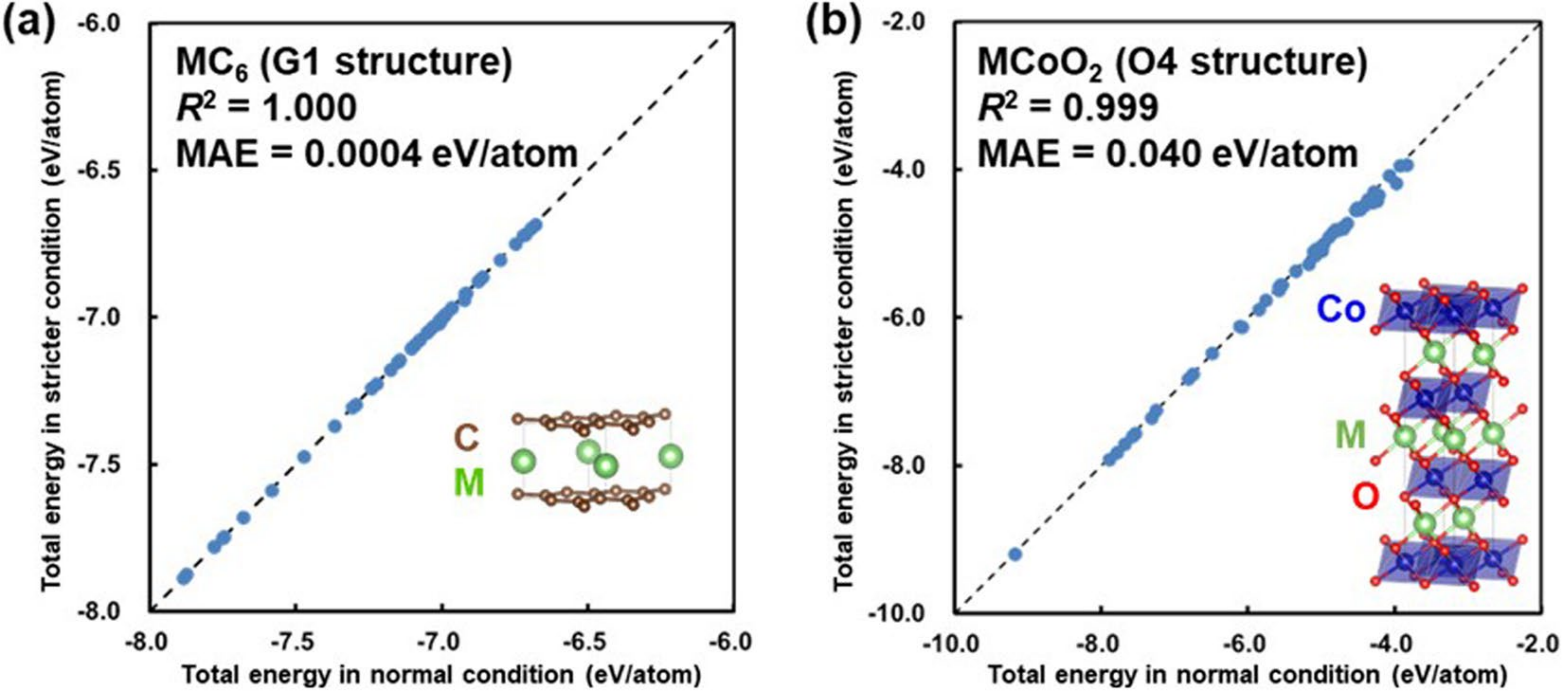
Comparison of total energy in the first-principles calculation with different energy convergence thresholds. The normal condition, 10^−4^ eV, was used for this study, and the stricter condition, 10^−6^ eV, was compared for validation. The materials are (**a**) MC_6_ (G1-structure in Fig. 2) and (**b**) MCoO_2_ (O4-structure in Fig. 3), with 48 intercalants M described in Fig. 7.

For the vdW force correction, as indicated in the Methods section, the correction method that produces the calculation results closest to the experimental values in the GIC has been adopted and is considered to be suitable for this system as shown in Table 1. Additionally, regarding the energy convergence criteria for electronic structure optimization, while 10^−4^ eV is not strict, calculations with a stricter convergence criterion of 10^−6^ eV have been performed for some compounds, confirming that the error is sufficiently small to be negligible as shown in Fig. 19.

Furthermore, in this study, the DFT+U method, which adds the on-site Coulombic interaction of localized electrons in compounds containing *d*-metals in the form of the generalized Hubbard model^90^, was not adopted. This decision was made because different values of *U* have been reported depending on the type of transition metals, its oxidation states, and the compounds, and arbitrarily selecting *U* as a parameter could bias the database. Therefore, it was considered to be preferable to perform calculations for all compounds without the inclusion of *U*.

### Simulated optimized structure

**Fig. 20:**
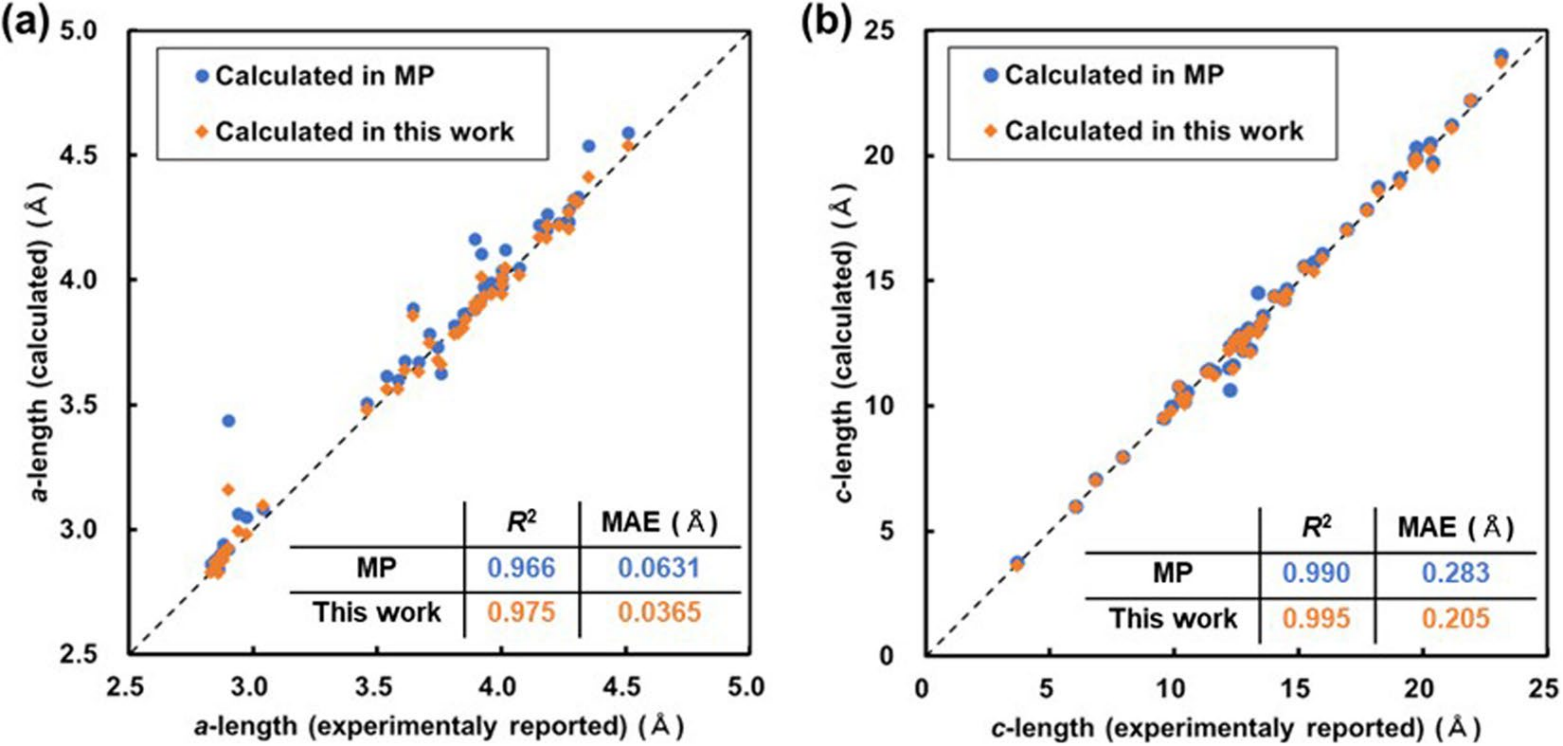
Comparison of lattice constants between experimentally reported values^66,67,91–132^ and those calculated in MP^22^ and in this study. (**a**) *a* - length, (**b**) *c* - length.

For structural optimization calculations, the energy convergence threshold for electronic state was set to 10^−4^ eV as mentioned above, and the residual force convergence threshold was set at 0.05 eV/Å. This condition is also not so strict. Therefore, to verify the validity of the optimized structures in this database, lattice constants (*a*, *c*) extracted from experimentally reported data^66,67,91–132^ and simulated in this study were compared in 50 layered intercalation compounds. The results are shown in Fig. 20. Additionally, to compare the validity of this database, results from structures computed in MP^22^ were also included for comparison. Because vdW correction was considered in this study and not considered in the MP, it can be observed that both the *R*^2^ and mean absolute error (MAE) values indicate that the accuracy of reproducing experimentally reported lattice constants is higher in this study compared to the MP. Therefore, it can be concluded that the database exhibits practical accuracy suitable for use as a structural database.

### Band structures and band gaps

The vdW correction method used in this study, rev-vdW-DF2, is not specifically designed for band structure or band gap corrections. Therefore, it must underestimate the band gap, as commonly pointed out in GGA(PBE)^133^. Actually, LiCoO2 (O4-type) is simulated to be 1.16 eV in this study, although it was simulated about 3 eV in GW calculation^134^. Regarding this issue, calculation methods known for accurately reproducing band gaps, such as HSE06^135^ or GW approximation^136^, are avoided due to their high computational cost. For instance, methods proposing the prediction of HSE06-level band gaps from GGA(PBE)-level calculations using machine learning techniques have been suggested^26^, and it is considered that applying these methods can yield values closer to the true ones (Table 2).

**Table 2:** Options for plotting band structure with “plot_bands.py”.

Option	Description
-h, --help	show this help message and exit
-e [ELEMENT_LIST …], --element_list [ELEMENT_LIST …]	element list for which you want to color the contribution as list, e.g. [“Ca1”] If there are same elements in the structure, please input the number of the element in POSCAR for example, [“Ag1”,”Ag2”]. If there is no number, it will be set to default number 1. If [“None”] is specified, it becomes single color plot. Default = [“None”]
-o [{s,px,py,pz,dxy,dyz,dxz,dz2,dx2-y2,all} …], --orbital_list [{s,px,py,pz,dxy,dyz,dxz,dz2,dx2-y2,all} …]	orbital list, if you want to plot all orbitals, please provide ‘all’ or nothing. Default = []
-s {up,down,both}, --spin {up,down,both}	spin for band plot, ‘up’ ‘down’ or ‘both’, default = ‘up’
--fig_size	figure size, (width, height), default = (24,8)
--ymin	ymin for band plot in unit of eV, default = −6
--ymax	ymax for band plot in unit of eV, default = 6
--src_dir	source directory, default = ‘for_figshare’
--dst_dir	destination directory, default = ‘.’

## Usage Notes

Modules in pymatgen^137^ are available for analyzing crystal structures, plotting band structures, and density of states (DOS) from VASP input files and output files (“vasprun.xml”). The python script “plot_bands.py” was provided by us on figshare^89^ also utilize pymatgen^137^ and it can describe band structure in various ways. We encourage readers to consider using these resources as references for tasks such as extracting band structures and exploring applications in machine learning.

In “plot_bands.py”, various functions useful for analysing band structure have been implemented. For example, there are following options when describing band structure:

To plot band structure with “plot_bands.py”, please make sure that database folder and “***-database.xlsx” file described in Data records are in source directory.

For example, if you want to plot the band structure of LC-824 CaC6_G3, there are some options.

If you want to plot only the band structure (up spin), you can input as follows:

*python plot_bands.py LC-824 -s up*

If you want to color all Ca-orbital projections:

*python plot_bands.py LC-824 -e Ca1 -o all -s up*

If you want to color Ca-s, dz2-orbitals projections:

*python plot_bands.py LC-824 -e Ca1 -o s dz2 -s up*

If you want to color all C-pz-orbital projections:

*python plot_bands.py LC-824 -e C1 C2 C3 C4 C5 C6 -o pz -o pz -o pz -o pz -o pz -o pz -s up*

If you want to plot band structure of basic *k*-path Hinuma *et al*.^73^:

*python plot_bands.py LC-824-primitive*

Instructions regarding the required environment and argument settings are available in the “readme_for_python_script.txt” file in figshare.

## Data Availability

We performed first-principles calculations with VASP software version 6.3.0, which is paid for and can only be accessed if purchased. However, making input files and analysing output files can perform by “pymatgen” module^137^.
